# Supplementary material for: Heterochronic parabiosis uncovers AdipoR1 as a critical player in retinal rejuvenation
Source: Sci Adv. 2025 Jul 16;11(29):eadv6642. doi: 10.1126/sciadv.adv6642 (PMC12266120; doi:10.1126/sciadv.adv6642)
Supplement: 20250716-1 [file sciadv.adv6642.v1.pdf]

HEALTH AND MEDICINE

Heterochronic parabiosis uncovers AdipoR1 as a critical player in retinal rejuvenation

Yidan Liu[1]†, Xiuxing Liu[1]†, Jianjie Lv[1]†, Qi Zhang[1]†, Zhenlan Yang[1], Xuhao Chen[1], Chenyang Gu[1], Chun Zhang[2], Yehong Zhuo[1]*, Wenru Su[1,3]*

Copyright © 2025 The Authors, some rights reserved; exclusive licensee American Association for the Advancement of Science. No claim to original U.S. Government Works. Distributed under a Creative Commons Attribution License 4.0 (CC BY).

Aging induces substantial structural and functional decline in the retina, yet the molecular drivers of this process remain elusive. In this study, we used heterochronic parabiosis (HP) combined with single-cell RNA sequencing to generate comprehensive transcriptomic profiles of murine retinas from young, aged, and HP pairs, aiming to identify antiaging targets. Our analysis revealed extensive transcriptional alterations across retinal cell types with aging. HP experiments demonstrated that systemic factors from young mice rejuvenated aged retinas and alleviated senescent phenotypes, while aged blood accelerated aging in young mice. Integrative analysis pinpointed adiponectin receptor 1 (AdipoR1) and the downstream adenosine 5′-monophosphate–activated protein kinase (AMPK) signaling pathway as central to the molecular mechanisms underlying retinal rejuvenation. Treatment with the AdipoR1 agonist AdipoRon reversed retinal aging. Mechanistically, AdipoR1-AMPK activation promoted mitochondrial function, contributing to the restoration of youthful cellular phenotypes. Together, our study identifies AdipoR1 as a therapeutic target for retinal aging and provides insights into the molecular programs driving retinal rejuvenation.

INTRODUCTION

Aging is a universal process characterized by the gradual decline of various bodily functions over the lifespan (*1*). The retina, a thin layer of neural tissue at the back of the eye, is composed of various cell types, including neurons, retinal pigment epithelium (RPE), and glial cells. Together, these components enable the retina to perform its critical role in capturing light and transmitting visual information to the brain, making it indispensable for vision and the overall quality of life. Aging exacerbates oxidative stress and chronic inflammation, accelerating visual dysfunction and increasing the risk of age-related retinal disorders, such as macular degeneration, diabetic retinopathy, and glaucoma—leading causes of irreversible vision loss and blindness (*2*, *3*). The rising prevalence of these conditions, coupled with their substantial social and economic burden (*4*), underscores the urgent need to elucidate the mechanisms driving retinal aging and to develop effective antiaging strategies to preserve vision.

In our previous research, we investigated retinal aging from the perspective of cellular senescence, revealing that senescent cells play a pathological role in this process (*5*). While this finding deepened our understanding, it also raised the question of how systemic and molecular factors contribute to retinal aging. Increasing evidence from human studies suggests that systemic changes, such as inflammation (*6*, *7*), metabolic dysfunction (*8*, *9*), and vascular alterations (*10*), play a crucial role in age-related retinal decline. These findings underscore the need to explore systemic interventions that may mitigate retinal aging.

Heterochronic parabiosis (HP) involves surgically connecting the circulatory systems of a young mouse and an aged mouse, allowing the exchange of blood-borne factors between them. This model provides a powerful experimental framework for investigating how exposure to youthful systemic factors can reverse aging through and how aging-related molecules in the bloodstream affect younger organisms. Early studies have shown that young blood could mitigate age-related decline in various tissues, including muscle, heart, liver, and brain, while aged blood accelerates aging phenotypes in young mice (*11*–*17*). Although some rejuvenating factors have been identified (*18*–*20*), the underlying cellular mechanisms and the systemic targets of these factors remain only partially understood.

Here, we investigated the rejuvenating effects of young circulation on the aged retina and elucidated the underlying mechanisms. Using high-throughput single-cell RNA sequencing (scRNA-seq), we constructed a comprehensive retinal cell atlas of young and aged mice. HP was used to identify key rejuvenating regulators and signaling pathways in the retina. Exposure to a youthful systemic environment notably alleviated neuroinflammation and reduced the senescence burden in the aged retina. Integrative bioinformatics analysis further identified adiponectin receptor 1 (AdipoR1) as a pivotal proyouth player. Oral administration of the AdipoR1 agonist AdipoRon (AR) mitigated both aging-related and senescent phenotypes, confirming the rejuvenating role of AdipoR1. Mechanistically, AdipoR1 activation restored adenosine 5′-monophosphate–activated protein kinase (AMPK) phosphorylation and enhanced mitochondrial function, including the restoration of mitochondrial membrane potential, promotion of mitophagy flux, and reduction of mitochondrial oxidative stress. The revitalization of mitochondrial dynamics supported the restoration of youthful cellular phenotypes and retinal function. Collectively, our findings identify AdipoR1 as a key regulator of retinal aging and highlight its potential as a therapeutic target for rejuvenation treatments.

RESULTS

Aging induces transcriptional alterations in multiple cell types of the retina

scRNA-seq represents a state-of-the-art technique for comprehensively analyzing tissue heterogeneity and uncovering intricate molecular mechanisms with unparalleled resolution and sensitivity. To

[1]State Key Laboratory of Ophthalmology, Zhongshan Ophthalmic Center, Guangdong Provincial Key Laboratory of Ophthalmology and Visual Science, Sun Yat-sen University, Guangzhou 510060, China. [2]Department of Ophthalmology, West China Hospital, Sichuan University, Chengdu, 610041, China. [3]Department of Ophthalmology, Shanghai Key Laboratory of Orbital Diseases and Ocular Oncology, Shanghai Ninth People's Hospital, Shanghai Jiao Tong University School of Medicine, Shanghai 200011, China.
*Corresponding author. Email: suwenru@sjtu.edu.cn (W.S.); zhuoyh@mail.sysu.edu.cn (Y.Z.)
†These authors contributed equally to this work.

investigate the transcriptional dynamics during retinal aging, we performed scRNA-seq analysis on retinal cells from young (6- to 8-week-old) and aged (20- to 22-month-old) mice.

By analyzing the expression pattern of specific marker genes, we identified 11 cell lineages as previously reported (Fig. 1A, fig. S1A, and table S1) (*5*). Differential expression gene (DEG) analysis was then conducted to determine the cell type–specific DEGs. RPE exhibited the highest number of DEGs followed by microglia and retinal ganglion cells (RGCs) (Fig. 1B), indicating that these cell populations were particularly affected by aging. These findings align with prior study (*21*).

We next explored the functional implications of these aging-associated DEGs. In retinal neurons, up-regulated DEGs were enriched in biological pathways related to DNA damage, oxidative stress, apoptosis, and impaired neuronal function (Fig. 1C). Conversely, down-regulated DEGs were predominantly associated with Gene Ontology (GO) terms related to neuronal homeostasis and function (fig. S1B). These findings indicate that key neuronal processes, including neurotransmitter transport, synaptic transmission, ion signaling, and the development and growth of the neural retina, are markedly impaired with aging.

Next, we examined age-related changes in the RPE. GO analysis of up-regulated DEGs revealed increased oxidative stress, inflammation, angiogenesis, cell death, accumulation of toxic substances and amyloid-β (Aβ), and dysregulated lipid metabolism in the aged RPE (Fig. 1, D and E). Conversely, aging was associated with a decline in key RPE functions, including phagocytosis, photoreceptor support, melanin biosynthesis, and the maintenance of the outer blood-retinal barrier (Fig. 1F). In addition, the down-regulation of GO terms related to mitochondrial function and NADH [reduced form of nicotinamide adenine dinucleotide (oxidized form)] regeneration suggested disrupted redox balance and a compromised antioxidant defense system in the RPE.

We then analyzed Aged-DEGs in retinal microglia and observed an up-regulation of several disease-associated microglia (DAM) signatures, including *Apoe*, *Tyrobp*, *Cd63*, *Cstb*, *Cstd*, and *Spp1* (Fig. 1G). GO analysis of up-regulated Aged-DEGs highlighted dysregulated cytokine production, increased activation, and cellular senescence in aged microglia (Fig. 1H), which was further validated by flow cytometry (FC; Fig. 1I). Correspondingly, transcription factor (TF) analysis revealed that ATF3, JUN, FOS, and BHLHE40 exhibited the highest regulon activities in the aged group. These TFs are known to play key roles in inflammatory regulation and microglial neuroprotection (Fig. 1J) (*22*–*25*). Collectively, aging promotes a pro-inflammatory phenotype, disrupts neuroprotective responses, and impairs microglial homeostasis, potentially contributing to pathological conditions. We further characterized the senescence phenotype in young and aged retinas. p16 expression was absent in young retinas; however, aged retinas exhibited clear p16 expression in microglia, vascular endothelial cells, and neurons (fig. S1C), indicating that senescence is broadly present across multiple retinal cell types.

Last, we examined cell type–specific DEGs implicated in age-related retinal diseases, including age-related macular degeneration (AMD), diabetic retinopathy, and glaucoma (Fig. 1, K to O). *Apoe* emerged as a central hub gene across multiple cell populations, while the RPE exhibited the highest number of disease-associated DEGs. Although the RPE and photoreceptors are primarily affected in AMD, dysregulated risk gene expression was observed across various cell types in the aged retina. While not all transcriptomic changes necessarily translate to functional consequences, this finding suggests that other cell types may contribute to disease progression through direct or indirect mechanisms, such as intercellular communication, offering mechanistic insights. To conclude, aging induces profound changes of the transcriptional landscape across various retinal cell types.

HP modulates both aging and senescence phenotypes in the retina

Blood-borne circulating factors are known to influence organ function during physiological aging. Therefore, we aimed to investigate the impact of HP on the retina of aged and young mice. To confirm the successful blood circulation of the HP model, we surgically connected a wild-type mouse (C57BL/6J) with a red fluorescent protein transgenic mice (Lyz2-Cre/Ai9) following established protocols (fig. S2, A and B) (*12*). Next, we constructed heterochronic parabionts between 18- to 20-month-old [heterochronic aged (Ahet)] and 6- to 8-week-old [heterochronic young (Yhet)] mice, alongside isochronic pairs of age-matched isochronic young (Yiso) and isochronic aged (Aiso) controls (Fig. 2A). After 2 months, parabionts were detached to enable physiological measurements that are difficult to conduct in anastomosed mice (*26*). Retinal function was assessed via electroretinography (ERG). To exclude potential confounding effects of surgery-related stress, ERG was first performed on detached isochronic mice and their age-matched controls that had not undergone any surgical interventions. The results showed no statistically significant differences (fig. S2C), indicating that the surgery did not affect retinal function. Further analysis of separated heterochronic pairs revealed that HP reversed the age-associated visual decline as evidenced by improvements in scotopic a- and b-waves as well as photopic b-wave responses. Notably young retinas showed no significant functional impairment following exposure to aged circulation (Fig. 2B and fig. S2D).

Visual behavioral tests were then conducted on young and aged mice. However, no notable differences were observed between the groups in either the cliff test or looming visual stimulus test (fig. S2, E and F). Consequently, these tests were not performed on the detached parabionts.

To investigate the structural changes induced by HP in the retina, we performed immunofluorescence (IF) staining. Aging causes dendritic extension of rod bipolar cells (RBCs) and horizontal cells (HCs) from the outer plexiform layer (OPL) into the outer nuclear layer (ONL) (*27*), a phenomenon that was reversed by HP in aged retinas and partially replicated in young retinas (Fig. 2, C and D). In addition, HP attenuated aging-related Müller glia activation, as evidenced by reduced glial fibrillary acidic protein (GFAP) immunoreactivity and thinner glial cell layers, although this effect was less pronounced in young glia (Fig. 2E). Furthermore, HP led to a reduction in microglial numbers in aged retinas, with the opposite trend observed in young parabionts (Fig. 2F).

In addition to modulating aging phenotypes, HP has recently been recognized for its role in regulating the senescence state in multiple tissues (*13*, *14*, *28*). Our previous research revealed an increased cellular senescence burden in the retina during aging (*5*). To investigate the impact of HP on retinal senescence, we quantified the expression of senescence and senescence-associated secretory phenotype (SASP) markers at both the RNA and protein levels. Quantitative polymerase chain reaction and FC analyses demonstrated a significant reduction in senescence and SASP burden in the aged

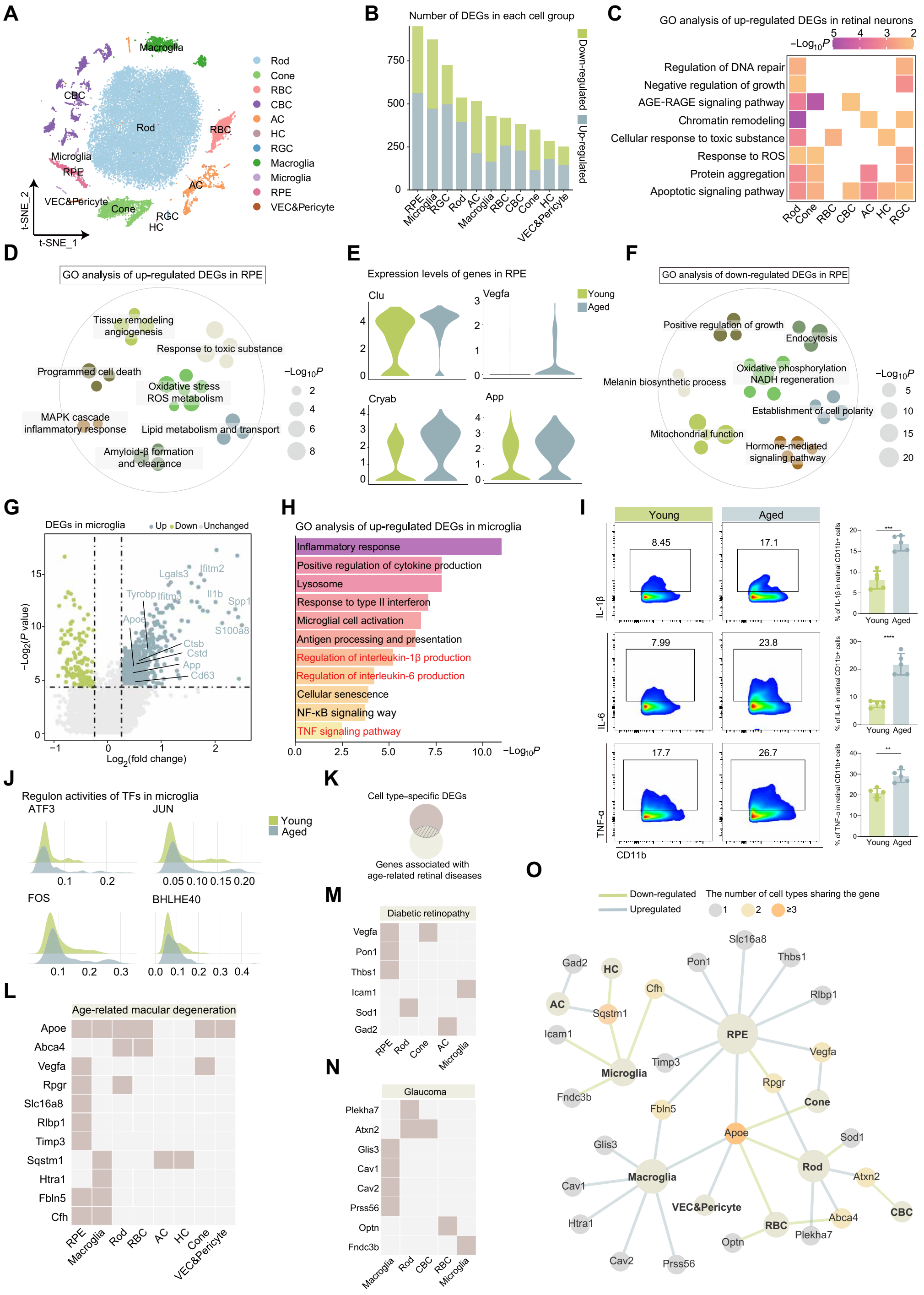

Fig. 1. Aging causes changes in gene expression across various retinal cell types. (**A**) t-SNE plot showing the distribution of different retinal cell types. (**B**) Bar plot showing the number of up-regulated and down-regulated DEGs in each cell group. (**C**) Heatmap showing the GO analysis of up-regulated DEGs in aged retinal neurons. AGE-RAGE, advanced glycation end product-receptor of advanced glycation end products. (**D**) Dot plot showing the GO analysis of up-regulated DEGs in the aged RPE, with each dot representing a GO term. Similar entries were clustered together. (**E**) Violin plots showing the expression levels of the indicated genes in the young RPE and aged RPE. (**F**) Dot plot showing the GO analysis of down-regulated DEGs in the aged RPE, with each dot representing a GO term. Similar entries were clustered together. (**G**) Volcano plot showing up-regulated and down-regulated DEGs in aged microglia. (**H**) Bar plot showing the functional analysis of up-regulated DEGs in aged microglia. (**I**) FC histograms (left) and column charts (right) showing the level of IL-1β, TNF-α, and IL-6 in the microglia (CD11b+) between young and aged retinas ($n = 5$ per group). (**J**) Ridge plots showing the regulon activities of the indicated TFs between young and aged microglia. (**K**) Venn diagram showing the integrative analysis of cell type–specific DEGs and genes associated with age-related retinal diseases. (**L** to **N**) Tile plots showing the distribution of overlapping genes associated with age-related macular degeneration (L), diabetic retinopathy (M), and glaucoma (N) across multiple retinal cell types. (**O**) Network diagram showing the interaction between the overall set of overlapping genes associated with age-related retinal diseases and various retinal cell types. Data are shown as the means ± SD. *P* values were analyzed using unpaired two-tailed Student's *t* test (I). $^{**}P < 0.01$, $^{***}P < 0.001$, and $^{****}P < 0.0001$.

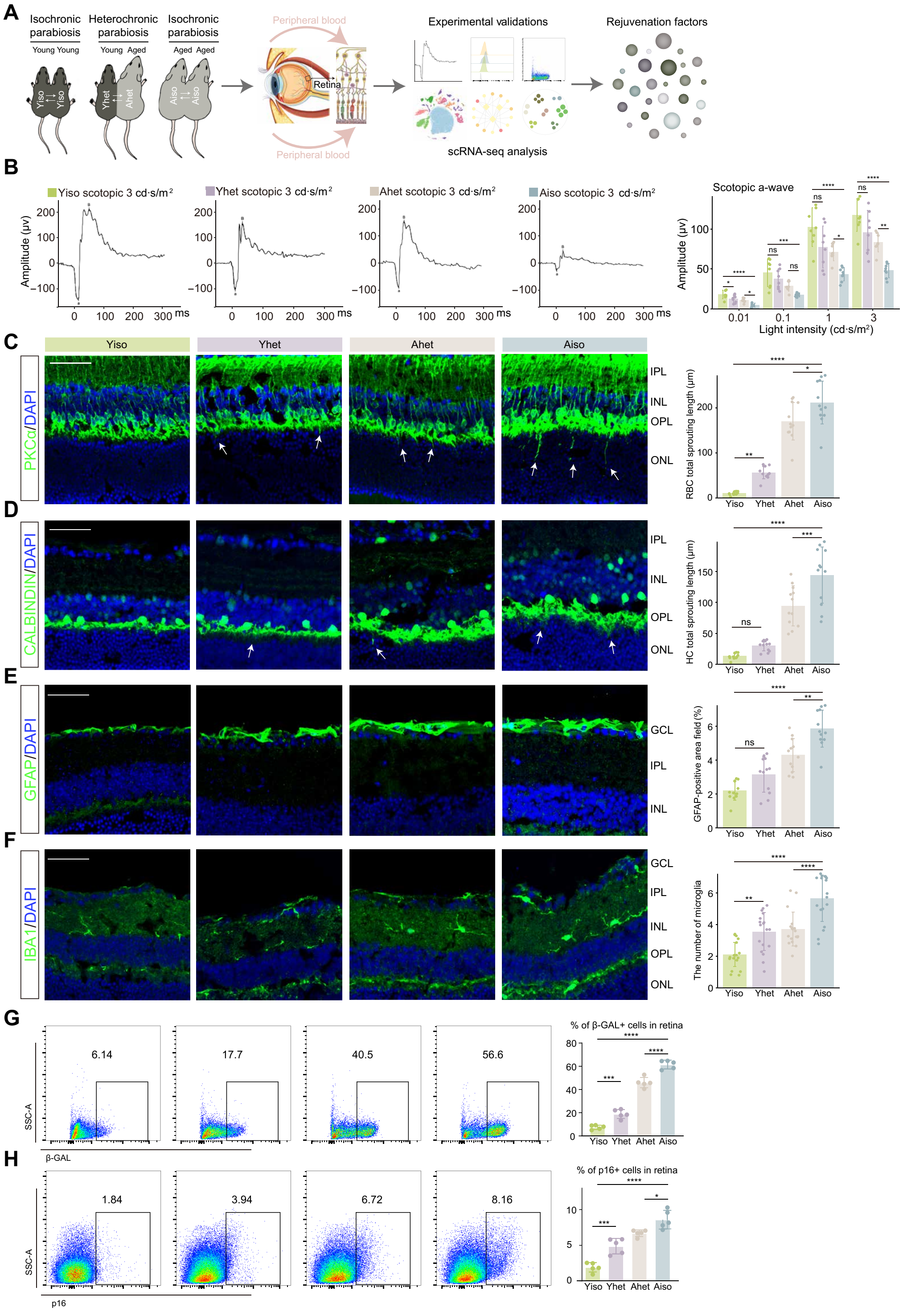

Fig. 2. HP modifies both the aging process and senescence characteristics of the retina. (**A**) Schematic diagram showing the study design and workflow. (**B**) Representative murine scotopic ERG at a light density of 3 cd·s/m^2 (left) and quantification bar charts of a-wave (right) across different light densities in the indicated groups. (**C** to **F**) Representative confocal images of retinal frozen sections of Yiso (far left), Yhet (second from the left), Ahet (center), and Aiso (second from the right) mice and bar charts of quantification (far right, $n = 6$ per group). Frozen sections are labeled with PKCα (C), calbindin (D), GFAP (E), and IBA1 (F). Arrows indicate the abnormal sprouting dendrites of RBCs (C) and HCs (D), which extend beyond the OPL into the ONL. Scale bar, 50 μm. (**G** and **H**) FC histograms (left) and column charts (right) showing β-GAL (G) and p16 (H) levels in retinal cells of the indicated groups ($n = 5$ per group). SSC-A, side scatter area. Data are shown as the means ± SD. *P* values were analyzed using one-way ANOVA with Bonferroni post hoc test [(B) to (H)]. ns, nonsignificant; $*P < 0.05$, $**P < 0.01$, $***P < 0.001$, and $****P < 0.0001$.

retina as well as reduced inflammatory microglia following HP (Fig. 2, G and H, and figs. S3, A to F, and S4, A to C). Conversely, the young retina exhibited a senescence response after exposure to aged blood circulation. In summary, HP effectively reprograms both the aging and senescence phenotypes of the retina, rejuvenating the aged retina to a more youthful state while inducing premature aging characteristics in the young retina.

HP reprograms the transcriptomic landscape of multiple cell types in the retina

To construct a comprehensive atlas of retinal tissue exposed to HP and elucidate the molecular mechanisms underlying its rejuvenating and proaging effects, we performed scRNA-seq analysis on retinal cells from both heterochronic and isochronic parabionts. We identified 11 major retinal cell lineages as previously described (Fig. 3A and table S2).

We first identified DEGs by comparing Aiso versus Yiso (Aiso-DEGs), Ahet versus Aiso (Ahet-DEGs), and Yhet versus Yiso (Yhet-DEGs). Given the well-established role of SASP factors in age-related chronic inflammation and degeneration in multiple tissues (*29*), we calculated SASP gene set (*30*) scores across these groups. The results indicated that HP alleviated the inflammatory response in the Ahet group while exacerbating SASP burden in the Yhet group, further supporting prior experimental findings. An integrative joint analysis of SASP signatures and up-regulated DEGs identified key genes, including Jun and Hmgb1 (Fig. 3B). The increased activity of these factors has been associated with inflammation, oxidative stress, and apoptotic cell death (*31*, *32*), potentially contributing to retinal aging and degeneration. In addition, we assessed retina homeostasis gene set scores. As expected, HP restored balance in aged retinas while reducing equilibrium in young retinas. Through comparative analysis of the retina homeostasis gene set and down-regulated DEGs, *Nxnl1* emerged as a hub gene (Fig. 3C). NXNL1 plays a crucial role in cellular redox regulation and antioxidant defense mechanisms, helping to maintain the reactive oxygen species (ROS) balance within retinal cells and protect against oxidative stress.

We next conducted functional enrichment analysis of Ahet- and Yhet-DEGs. In neurons, HP reversed the decline of intrinsic functions essential for proper retinal function and visual processing in the aged retina (Fig. 3D). Notably, BDNF (brain-derived neurotrophic factor) signaling, which is implicated in neuroprotection, was up-regulated. HP also alleviated the dysregulation of biological pathways in the retina during the aging process (fig. S1B and Fig. 3E). However, in young neuroretinas exposed to HP, we observed reduced synaptic signal processing and mitochondrial activity, as well as increased oxidative stress, endoplasmic reticulum stress, and cellular senescence (fig. S5, A and B). In the aged RPE of Ahet mice, HP enhanced various aspects of cellular function, metabolism, and structural organization (Fig. 3F). Notably, the adiponectin-activated signaling pathway and NADH regeneration were among the up-regulated GO terms, suggesting that HP may promote cellular energy metabolism and redox balance in the aged RPE. Meanwhile, HP alleviated pathways related to cell death, cytokine production, and inflammation in the aged RPE (Fig. 3G). Conversely, aged circulation dampened multiple pathways involved in maintaining proper RPE function and homeostasis in young RPE (fig. S5, C and D).

We then compared the responsiveness of various retinal cell types to HP treatment. Prorejuvenative DEGs (PR-DEGs) were defined as the intersection of Ahet-DEGs and Aged-DEGs, while progeronic DEGs (PG-DEGs) were defined as the intersection of Yhet-DEGs and Aged-DEGs (Fig. 3H). The percentage of PR-DEGs to Aged-DEGs reflected the rejuvenating effects of HP on the aged retina, whereas the percentage of PG-DEGs to Aged-DEGs indicated the proaging effects of HP on the young retina.

As shown in the bar plots, non-neuronal cell types, including RPE, vascular endothelial cells and pericytes (VEC&Pericytes), and microglia, were more sensitive to changes in the circulation milieu compared to neurons and macroglia (Fig. 3, I and J). Focusing on the RPE, the most responsive cell type to HP, we identified *Yy1* as the top regulator of PR-DEGs, with its activity the highest in the Yiso group, followed by Yhet, Ahet, and Aiso groups (Fig. 3, K and L). Pathway analysis of Yy1 target genes revealed that Yy1 played a crucial role in epigenetic modifications, NADH regeneration, autophagy, and tight junction maintenance, all of which are essential for proper RPE function (Fig. 3M). Furthermore, Yy1 has been implicated in the rejuvenation of hematopoietic stem and progenitor cells (*12*). In parallel, analysis of TFs regulating PG-DEGs indicated that Bhlhe40 was the primary factor driving proaging transcriptional regulation in the RPE (Fig. 3, N and O). Bhlhe40 modulates genes related to the inflammatory response and cytokine production, and its activity contributed to the up-regulation of epithelial-mesenchymal transition and endoplasmic reticulum stress, promoting the aging of young RPE when exposed to aged blood (Fig. 3P). To conclude, HP reshapes the transcriptional profile across various retinal cell types, with the RPE exhibiting the highest level of responsiveness to HP treatment.

Bioinformatic analysis identifies the critical rejuvenating player AdipoR1 in the retina

We next aimed to identify proyouthful regulators through single-cell data mining. First, we performed an integrative analysis of PR-DEGs from the whole retina with public aging-related gene sets including Aging Atlas, GenAge, and CellAge. We then assessed the frequency at which the intersecting genes also appeared as cell type–specific DEGs in the retina. The top two genes identified were *Gpx4* and *Adipor1* (Fig. 4A). Gpx4 (glutathione peroxidase 4) is an antioxidant enzyme that protects cells from oxidative stress and regulates ferroptosis. Correspondingly, functional analysis of rescued DEG involving Gpx4 highlighted a response to oxidative stress and protein-DNA complex organization (Fig. 4B). AdipoR1 is involved in metabolic regulation, with known antidiabetic, antiatherosclerotic, and antiaging properties (*33*). In the context of HP-induced retinal rejuvenation, AdipoR1 participates in AMPK signaling, the longevity-regulating pathway, and the integration of energy metabolism (Fig. 4C). Further functional analysis of up-regulated PR-DEGs revealed that HP most effectively restored signaling related to AMPK activation, including energy metabolism, mitochondrial biogenesis, autophagy, and cellular respiration (Fig. 4D). These findings highlight AdipoR1 as a more promising player in mediating the rejuvenating effect of HP in the aged retina.

We next examined the expression of AdipoR1 in the retina. As shown in the t-distributed stochastic neighbor embedding (t-SNE) plot, AdipoR1 is expressed across various retinal cell types (Fig. 4E). The heatmap and violin plot showed that the expression of AdipoR1 decreased with aging across all retinal cell types and was restored to varying extents by HP (Fig. 4F). Both AdipoR1 and AMPK signaling were reduced in the aged retina and were increased following HP as indicated by the scRNA-seq data (Fig. 4, G, H, and J). This was further confirmed at the protein level by FC and Western blotting (Fig. 4, I, K, and L, and fig. S5E).

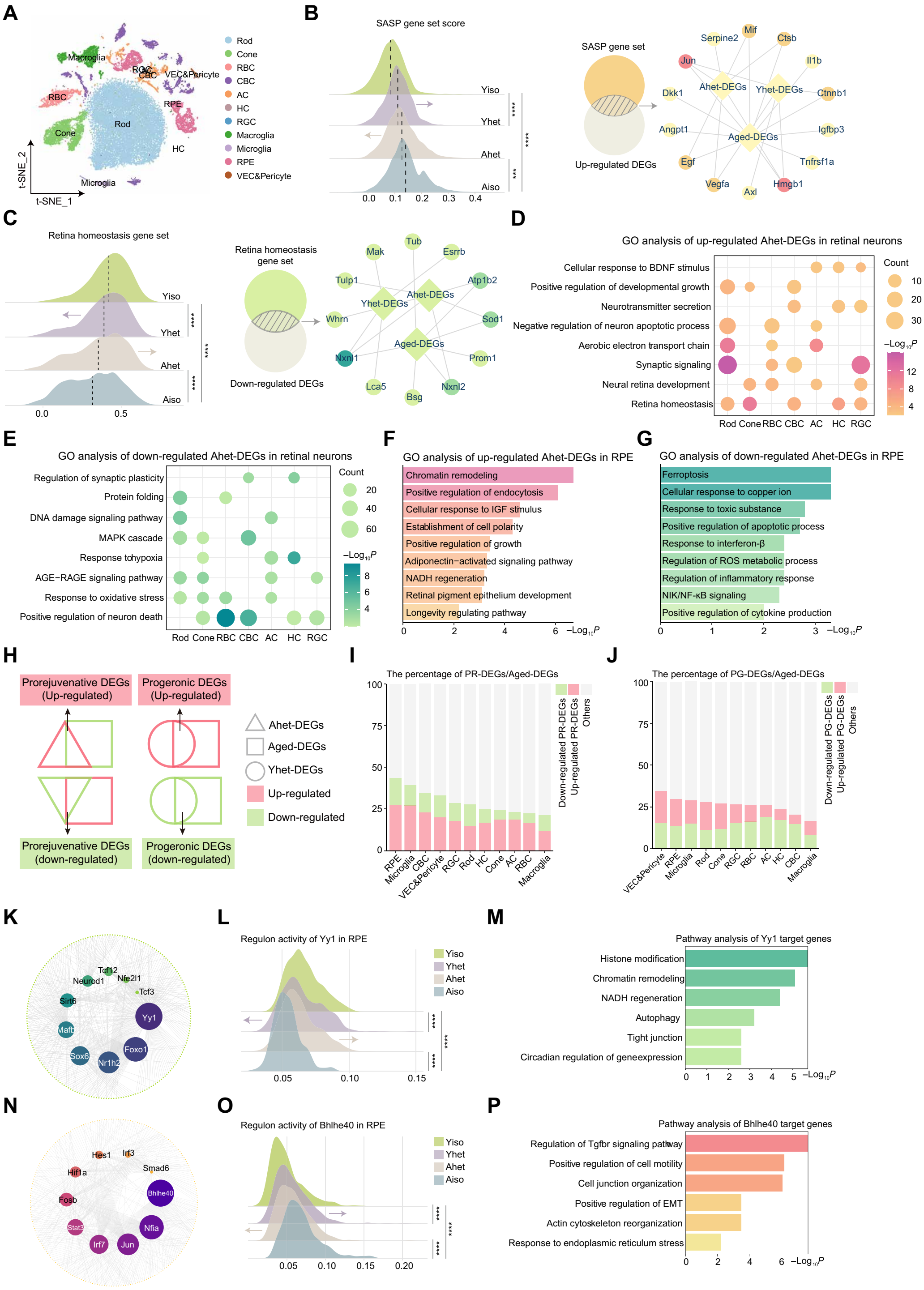

Fig. 3. HP reshapes the transcriptomic landscape across retinal cell types. (**A**) t-SNE plot showing the distribution of retinal cell types. (**B**) Ridge plot showing SASP gene set scores; Venn diagram showing the overlap between the SASP gene set and up-regulated DEGs; network diagram showing interactions between the overlapping SASP genes and DEGs. (**C**) Ridge plot showing retina homeostasis gene set scores; Venn diagram showing the overlap between the retina homeostasis gene set and up-regulated DEGs; network diagram showing interactions between the overlapping retina homeostasis genes and DEGs. (**D** and **E**) Dot plot showing the GO analysis of up-regulated and down-regulated Ahet-DEGs across retinal neurons. (**F** and **G**) Bar plot showing the GO analysis of up-regulated and down-regulated Ahet-DEGs in the RPE. (**H**) Venn diagram showing the definition of up-regulated and down-regulated prorejuvenative and progeronic DEGs. (**I** and **J**) Bar plot showing the percentage of PR-DEGs and PG-DEGs relative to Aged-DEGs. (**K**) Network plot showing the interaction between the TFs and target genes. The size of the dot indicates the number of target genes. (**L**) Ridge plot showing the regulon activity of Yy1 in the RPE across the indicated groups. (**M**) Bar plot showing the pathway analysis of Yy1 target genes. (**N**) Network plot showing the interaction between the TFs and target genes. (**O**) Ridge plot showing the regulon activity of Bhlhe40 in the RPE across groups. (**P**) Bar plot showing the pathway analysis of Bhlhe40 target genes. *P* values were analyzed using two-sided Wilcoxon rank-sum tests with Benjamini-Hochberg correction to adjust for multiple testing [(B), (C), (L), and (O)]. ****P* < 0.001 and *****P* < 0.0001.

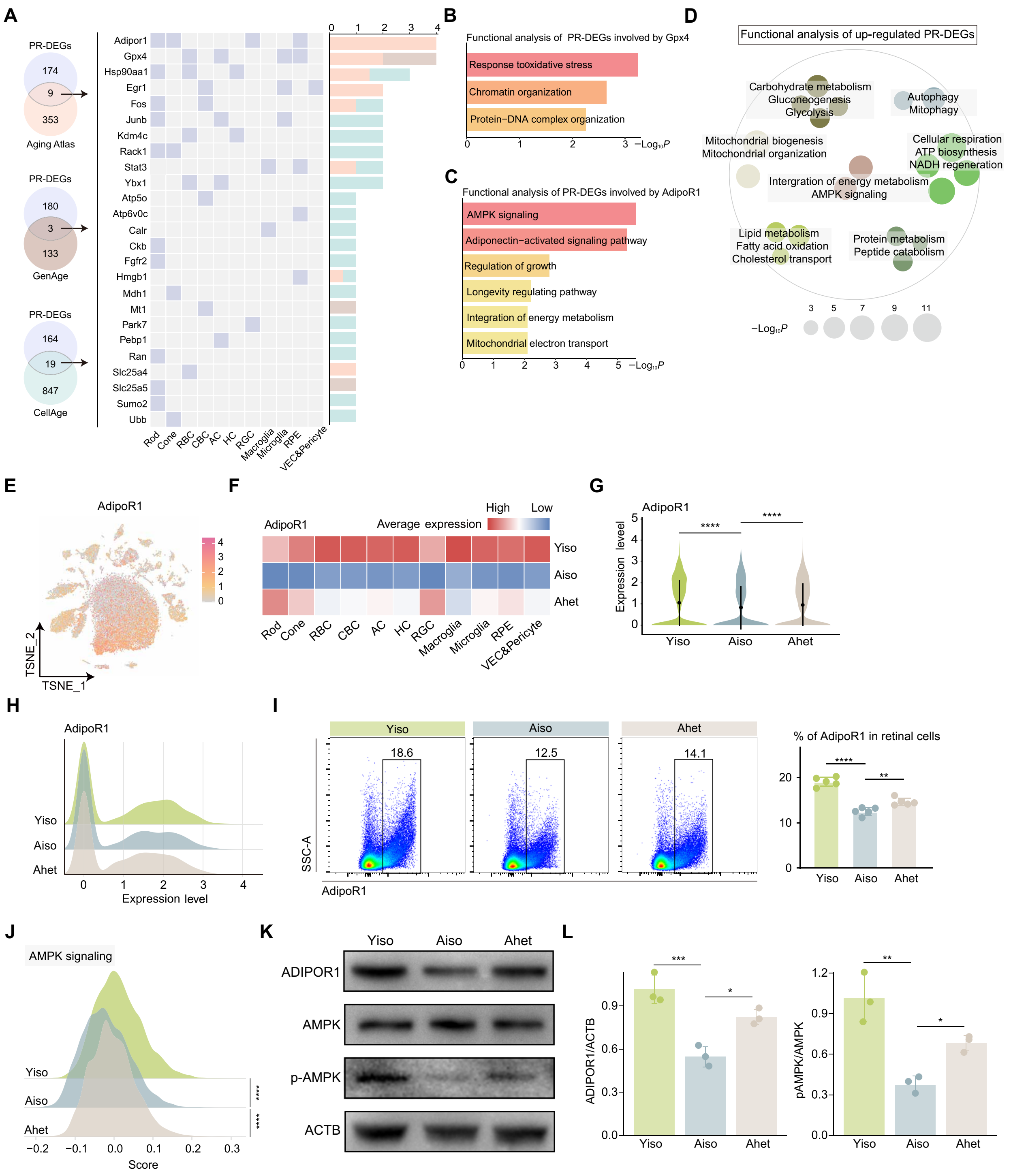

Fig. 4. Bioinformatic analysis reveals AdipoR1 as a key rejuvenating factor in the retina. (**A**) Venn diagram (left) showing the overlap between PR-DEGs and datasets including Aging Atlas, GenAge, and CellAge. Tile plot (middle) showing the distribution of overlapping genes across various retinal cell types. Bar plot (right) showing the frequency of overlapping genes. (**B**) Bar plot showing the functional analysis of PR-DEGs involved with Gpx4. (**C**) Bar plot showing the functional analysis of PR-DEGs involved with AdipoR1. (**D**) Dot plot showing the functional analysis of up-regulated PR-DEGs, with each dot indicating a GO term. Similar entries were clustered together. (**E**) t-SNE plot showing the expression of AdipoR1 in retinal cells. (**F**) Heatmap showing the average expression of AdipoR1 across various retinal cell types of the indicated groups. (**G**) Violin plot showing AdipoR1 expression levels across the indicated groups. (**H**) Ridge plot showing AdipoR1 expression levels across the indicated groups. (**I**) FC histograms (left) and column charts (right) showing AdipoR1 levels in retinal cells across the indicated groups ($n = 5$ per group). (**J**) Ridge plot showing AMPK signaling gene set scores across the indicated groups. (**K**) Immunoblot analysis of the indicated protein levels across three groups. (**L**) Bar plot showing the quantification of AdipoR1 (left) and p-AMPK (right) of the immunoblot ($n = 3$ per group). Data are shown as the means ± SD. *P* values were analyzed using two-sided Wilcoxon rank-sum tests with Benjamini-Hochberg correction to adjust for multiple testing [(G) and (J)] or one-way ANOVA with Bonferroni post hoc test [(I) and (L)]. $*P < 0.05$, $**P < 0.01$, $***P < 0.001$, and $****P < 0.0001$.

In the physiological context, AdipoR1 is primarily activated by adiponectin, a hormone primarily secreted by adipose tissue that regulates energy metabolism and inflammation (*33*). To further investigate systemic changes, we measured circulating adiponectin levels by enzyme-linked immunosorbent assay (ELISA) and observed a significant decrease in the aged group (fig. S5F). However, adiponectin levels in Ahet mice did not show a notable increase compared to Aiso mice. Collectively, the combination of scRNA-seq analysis and experimental validation identified AdipoR1 as a potential rejuvenating player in the aged retina.

AR treatment relieves degeneration changes and reduces senescence burden in the aged retina

To further explore whether activation of AdipoR1-AMPK signaling could alleviate aging and senescence phenotypes of aged retina, we used AR, an agonist of adiponectin receptors (*34*–*36*). We treated 18- to 20-month-old mice with or without AR mixed in a standard diet for 2 months. Following treatment, we performed ERG and IF staining to evaluate functional and structural changes in the retina. ERG measurements indicated that visual perception under scotopic conditions was rescued by AR (Fig. 5, A and B, and fig. S6A). Moreover, synaptic remodeling was also ameliorated in the AR-treated group as evidenced by a decrease in dendritic sprouts in RBCs and HCs (Fig. 5, C and D, and fig. S6, B and C). In addition, AR therapy reduced GFAP immunoreactivity in macroglia and the number of microglia in aged mice (Fig. 5, E and F, and fig. S6, D and E).

We further investigated the effects of AR treatment on cellular senescence. Quantitative polymerase chain reaction analysis confirmed a significant reduction in senescence and SASP-associated signatures upon AR treatment (Fig. 5G). These results were further supported by FC analysis at the protein level, which revealed a partial depletion of key senescence markers, including senescence-associated β-galactosidase (SA-β-GAL), p16, and p21, which accumulate during aging. Moreover, AR treatment led to decreased levels of several SASP cytokines, including interleukin-6 (IL-6), IL-1β, and tumor necrosis factor–α (TNF-α) (Fig. 5H). In conclusion, AR treatment effectively reverses age-associated functional impairment and degenerative morphological changes and relieves senescence burden in the aged retina.

AR treatment alleviates senescence and inflammation in retinal cells

After confirming the rejuvenating effects of AR treatment in the whole retina in vivo, we further explored its impact on specific retinal cell types using in vitro cell models and primary microglia. The 661W cell line, commonly used for retinal neurons, models photoreceptors, while Adult Retinal Pigment Epithelial cell line-19 (ARPE-19) cells are used to represent the RPE. To induce stress-related cellular senescence in 661W and ARPE-19, we used H_2O_2, establishing an in vitro senescent cell model (*5*). FC analysis revealed that AR treatment significantly reduced the expression of senescence-associated markers such as SA-β-GAL, p16, p53, and p21 across different cell types compared to the untreated senescent group (Fig. 6, A and B). These findings suggested that AR treatment could effectively alleviate the senescence burden in various retinal cell types.

We also isolated the primary retinal microglia and used FC to confirm the microglial identity (fig. S7A). Lipopolysaccharide (LPS) was used to induce a senescence phenotype in primary microglia (*37*). Similarly, AR treatment resulted in a marked decrease in senescence-associated markers and pro-inflammatory SASP cytokines, including IL-6, IL-1β, and TNF-α (Fig. 6, C and D), further demonstrating the anti-inflammatory properties of AR. In conclusion, AR treatment relieves the senescence burden and mitigates inflammation in different retinal cell types.

AdipoR1-AMPK activation relieves retinal aging partly through mitochondrial function improvement

After validating the revitalizing effects of AR both in vivo and in vitro, we aimed to explore the underlying molecular mechanisms. Western blot analysis (Fig. 7, A to C) confirmed that AR significantly upregulated AdipoR1 expression and increased the levels of phosphorylated AMPK, the active form, in aged retinal cells. AMPK is a key energy regulator, and mitochondria are central to cellular energy production. However, during aging, mitochondria undergo structural and functional changes across various tissues. Age-related declines in mitochondrial biogenesis and membrane potential (MMP) compromise adenosine 5′-triphosphate (ATP) synthesis and energy production. Impaired mitophagy and the accumulation of mitochondrial DNA (mtDNA) mutations further exacerbate oxidative stress, contributing to cellular dysfunction.

These observations prompted us to investigate how mitochondrial function is affected in the aged retina and whether AR-induced AdipoR1-AMPK activation could alleviate retinal aging, at least partially, by restoring mitochondrial function. To assess mitochondrial function, we measured mitochondrial oxidative stress using MitoSOX Red (MSR) and MMP using tetramethylrhodamine methyl ester (TMRM). Aging resulted in a marked increase in MSR fluorescence intensity and a decrease in TMRM intensity, indicating heightened oxidative stress and loss of membrane potential. Notably, AR treatment effectively reduced oxidative stress and restored MMP (Fig. 7, D and E). In the in vitro 661W cell line, AR produced similar effects on senescent cells (Fig. 7, F and G).

Mitophagy is crucial for maintaining mitochondrial quality, and mitophagy induction serves as a strategy to reduce age-associated inflammation and increase health span. Light chain 3II (LC3II) is a widely used marker of autophagy. IF staining showed that AR promoted mitophagy in 661W cells as evidenced by the increased colocalization of mitochondria and LC3II (Fig. 7H). Mitophagy flux was further quantitatively evaluated using FC, a novel method for this purpose (*38*). MitoTracker staining, combined with the mitophagy inducer CCCP (carbonyl cyanide *m*-chlorophenyl hydrazone) and the lysosomal inhibitor HCQ (hydroxychloroquine), allows for the determination of mitophagy flux in both cell lines and tissue. FC analysis revealed that AR markedly promoted mitophagy in aged retinal cells (Fig. 7I). Last, we measured the oxygen consumption rate (OCR) to assess the mitochondrial bioenergetic capacity, which revealed that AR enhanced basal respiration, ATP production, and maximal respiration in senescent 661W cells (Fig. 7, J and K). Parallel to the in vitro results, AR treatment also partially restored ATP content in retinal cells (Fig. 7L). In summary, AR treatment activates the AdipoR1-AMPK pathway in the aged retina, improving mitochondrial function and potentially underpinning its rejuvenating effects.

DISCUSSION

Vision is widely regarded as the most essential human sense. Age-related visual decline greatly affects the quality of life in older adults, limiting daily activities, social interactions, and independence. Retinal aging is a major risk factor for irreversible blinding diseases,

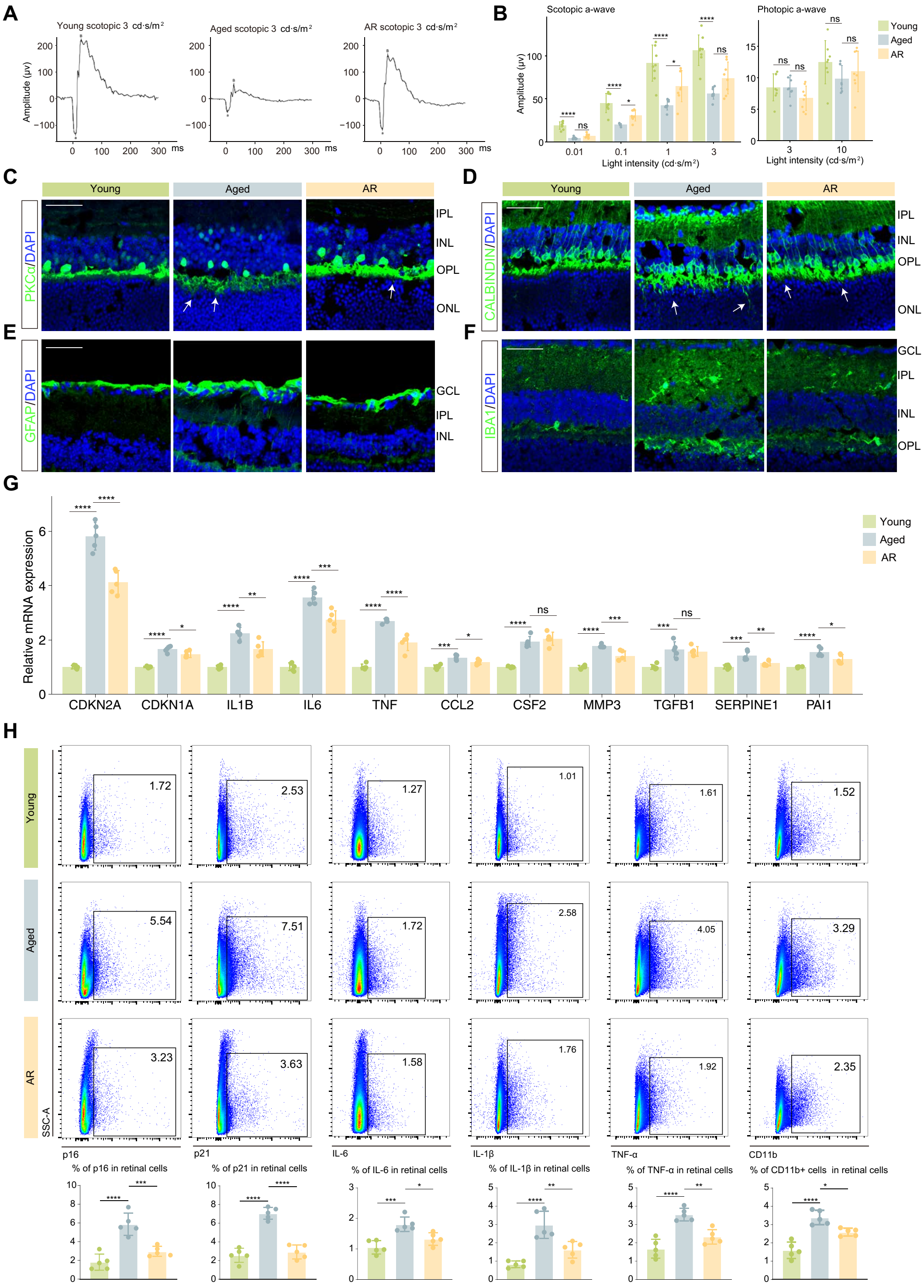

Fig. 5. AR treatment alleviates degenerative changes and lessens the senescence burden in the aging retina. (**A**) Representative murine scotopic ERG at a light density of 3 cd·s/m^2 across different groups. (**B**) Quantification bar charts of scotopic (left) and photopic (right) a-waves at different light densities across the indicated groups. (**C** to **F**) Representative confocal images of retinal frozen sections of young (left), aged (middle), and AR (right) mice ($n = 6$ per group). Frozen sections are labeled with PKCα (C), calbindin (D), GFAP (E), and IBA1 (F). Arrows indicate the abnormal sprouting dendrites of RBCs (C) and HCs (D), which extend beyond the OPL into the ONL. Scale bar, 50 μm. (**G**) Bar plot showing the relative mRNA expression of senescence markers and SASP factors across different groups ($n = 5$ per group). (**H**) FC histograms (top) and column charts (bottom) showing the level of p16, p21, IL-6, IL-1β, TNF-α, and CD11b in retinal cells of the indicated groups ($n = 5$ per group). Data are shown as the means ± SD. *P* values were analyzed using one-way ANOVA with Bonferroni post hoc test [(B), (G), and (H)]. *$P < 0.05$, **$P < 0.01$, ***$P < 0.001$, and ****$P < 0.0001$.

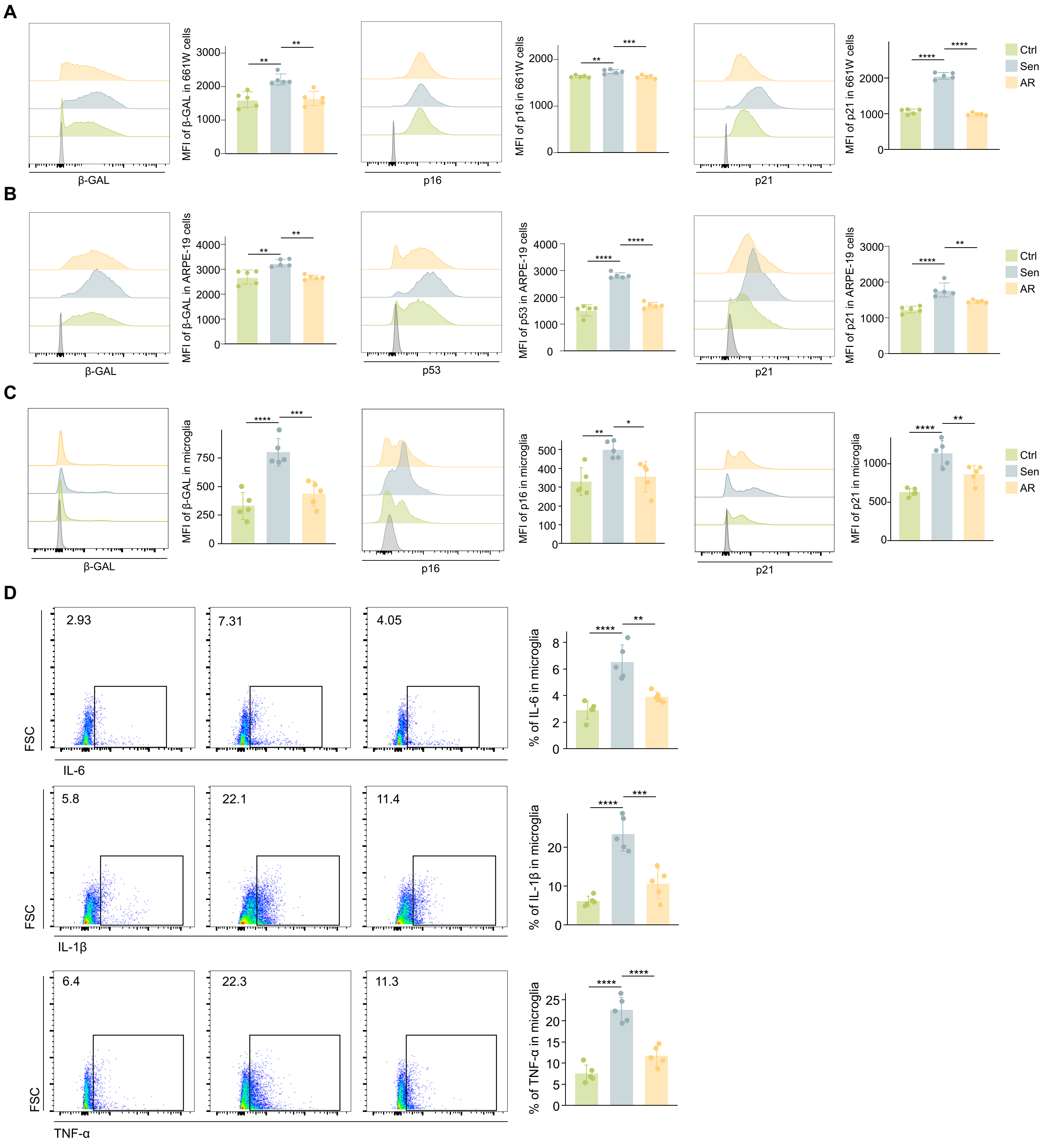

Fig. 6. AR treatment reduces the senescence burden and alleviates inflammation in retinal cell lines and primary microglia. (**A** to **C**) FC histograms (left) and column charts (right) showing the mean fluorescence intensity (MFI) of β-GAL (left), p16 (middle), p53 (middle), and p21 (right) in 661W (A) and ARPE-19 (B) cells and primary microglia (C) across the indicated groups ($n = 5$ per group). (**D**) FC histograms (left) and column charts (right) showing the level of IL-6, IL-1β, and TNF-α in primary microglia across the indicated groups ($n = 5$ per group). FSC, forward scatter. Data are shown as the means ± SD. *P* values were analyzed using one-way ANOVA with Bonferroni post hoc test [(A) to (D)]. $*P < 0.05$, $**P < 0.01$, $***P < 0.001$, and $****P < 0.0001$.

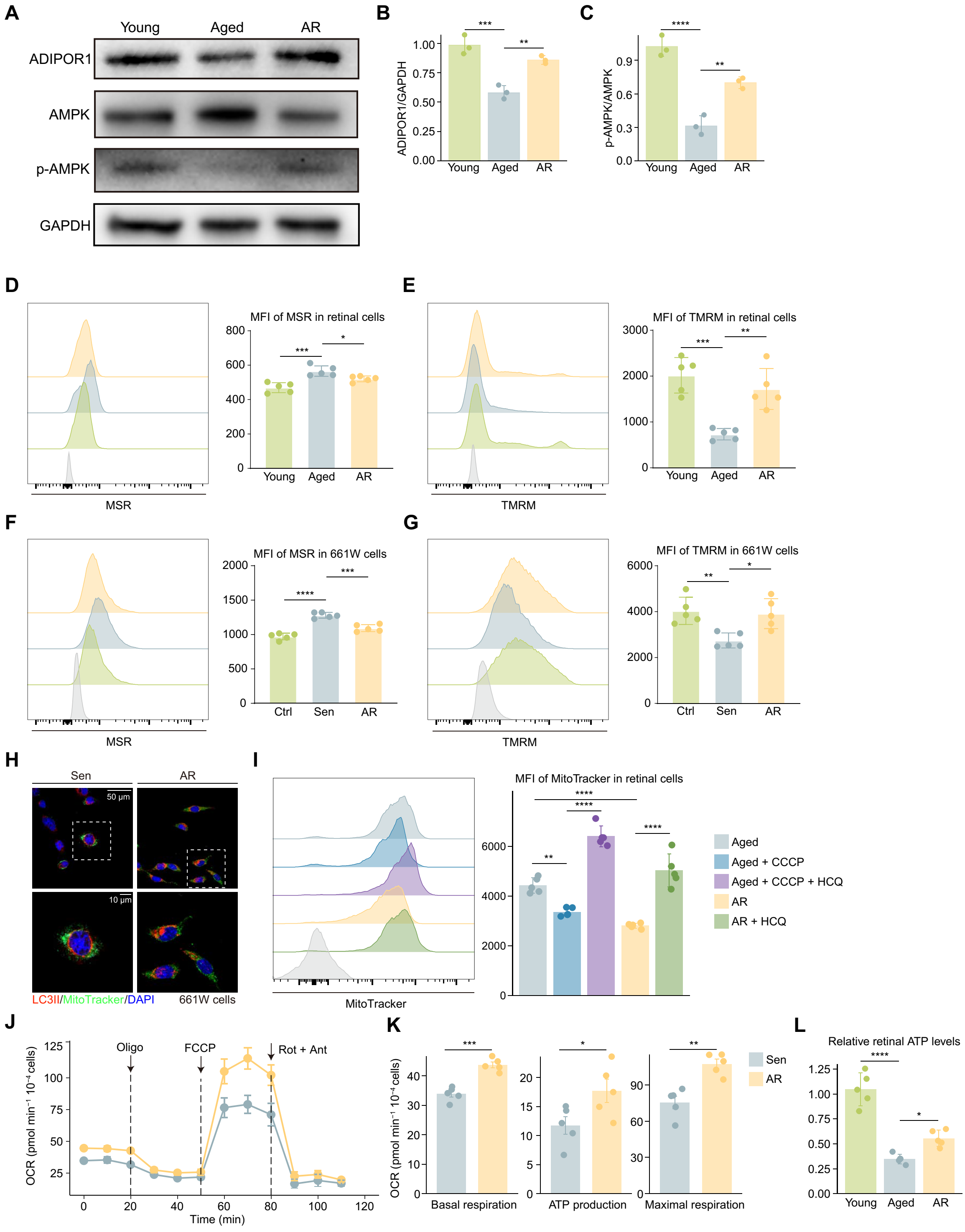

Fig. 7. AdipoR1-AMPK activation mitigates retinal aging in part by enhancing mitochondrial function. (**A**) Immunoblot showing the expression level of the indicated protein across three groups. (**B** and **C**) Bar plot showing the quantification of AdipoR1 (B) and p-AMPK (C) levels from the immunoblot ($n = 3$ per group). (**D** and **E**) FC histograms (left) and column charts (right) showing the MFI of MSR (D) and TMRM (E) in retinal cells across the indicated groups ($n = 5$ per group). (**F** and **G**) FC histograms (left) and column charts (right) showing the MFI of MSR (F) and TMRM (G) in 661W cells across the indicated groups ($n = 5$ per group). (**H**) Representative confocal images of 661W cells showing LC3II expression and mitochondrial morphology ($n = 6$ per group). (**I**) FC histograms (left) and column charts (right) showing the MFI of MitoTracker in retinal cells across the indicated groups ($n = 5$ per group). (**J**) OCR assessment of 661W cells from the indicated groups using a Seahorse XF24 Analyzer. Oligomycin (Oligo), FCCP, and rotenone and antimycin (Rot + Ant) were sequentially injected as specified. (**K**) Bar plot showing ATP production, basal respiration, and maximal respiration measurements in 661W cells. (**L**) Bar plot showing the relative retinal ATP levels across the indicated groups ($n = 5$ per group). Data are shown as the means ± SD. *P* values were analyzed using unpaired two-tailed Student's *t* test (K) or one-way ANOVA with Bonferroni post hoc test [(B) to (G), (I), and (L)]. $*P < 0.05$, $**P < 0.01$, $***P < 0.001$, and $****P < 0.0001$.

underscoring the urgent need to elucidate its mechanisms and develop effective antiaging interventions. Our study provides a detailed single-cell landscape of the retina under HP and identifies AdipoR1 as a key regulator of retinal aging through the integration of HP and scRNA-seq. Activation of AdipoR1 via AR effectively reversed aging-associated phenotypes by modulating the AMPK-mitochondria axis. These findings highlight the therapeutic potential of AdipoR1 and offer promising strategies to combat age-related retinal degeneration and preserve vision.

Retinal aging is a complex process characterized by progressive structural, cellular, and functional changes that contribute to visual decline. Key age-related alterations include increased oxidative stress, chronic low-grade inflammation, and impaired cellular homeostasis, all of which are implicated in retinal neurodegeneration. In this study, we comprehensively characterized retinal aging by examining several aspects, including the up-regulation of senescence and SASP markers, morphological alterations in RBCs and HCs, increased microglial infiltration, and functional impairments detected via ERG.

The aged retinal microglia exhibited a complex activation profile. The increased expression of DAM-associated genes along with the enrichment of the lysosome pathway suggested an enhanced phagocytic capacity. DAM represent a specialized microglial state observed under neurodegenerative conditions (*39*, *40*), particularly in Alzheimer's disease, where they help clear apoptotic cells and protein aggregates. While this up-regulation of phagocytosis-related genes might initially serve a protective role in maintaining retinal homeostasis, excessive or dysregulated phagocytic activity could lead to the unintended clearance of synapses and healthy neurons, contributing to age-related retinal neurodegeneration. Aged retinal microglia also showed a heightened inflammatory response, as evidenced by the up-regulation of *Il1b*, *Spp1*, *S100a8*, *Tnf*, and *Il6*. Persistent low-grade inflammation, known as inflammaging, has been implicated in age-related retinal diseases (*7*). In addition, increased expression of *Ifitm2* and *Ifitm3*, genes associated with type II interferon signaling, indicated a shift toward an activated immune state, potentially exacerbating inflammatory signaling cascades in the aged retina. A small population of interferon-responsive microglia has also been reported in the aged brain (*41*), further supporting this phenomenon. Together, these findings indicate that aged retinal microglia exhibit a mixed activation profile rather than a single uniform state. However, given that our sequencing data were obtained from whole retinal tissue, identifying distinct microglial subpopulations remains challenging because of the limited number of cells. Future study is expected to enrich retinal microglia before sequencing, allowing for deeper characterization of their heterogeneity and the identification of functionally distinct microglial states in the aged retina.

In aged retina, we observed up-regulation of genes associated with Aβ formation in RPE cells. However, because murine Aβ lacks critical residues necessary for aggregation, it is unlikely that functional Aβ deposits form in these mice. Instead, these gene expression changes might indicate alternative roles, such as intracellular stress responses, proteostasis regulation, or extracellular vesicle–mediated signaling in the aged RPE. In human aged retina and retinal diseases like AMD, Aβ accumulation has been detected in the RPE and drusen deposits, contributing to retinal degeneration (*42*–*44*). Further studies are needed to explore the functional significance of these gene changes in the murine RPE and determine whether they reflect conserved aging-related pathways relevant to human retinal pathology.

Despite these pronounced age-related changes at the molecular, cellular, and electrophysiological levels, behavioral tests assessing visually guided responses, such as the cliff test and looming visual stimulus test, did not reveal significant differences between young and aged mice. It is possible that compensatory neural mechanisms, such as cortical plasticity or an increased reliance on nonretinal visual processing, help maintain performance in these tasks despite retinal aging. In addition, behavioral tests often require a higher threshold of impairment to detect functional deficits, whereas ERG can capture subtle electrophysiological changes that may not immediately translate into noticeable behavioral alterations. Further studies incorporating more sensitive behavioral paradigms or longitudinal assessments may provide deeper insights into the functional consequences of retinal aging.

The HP model offers a robust experimental framework for uncovering key players driving aging and rejuvenation in a systemic manner. In our study, we further contributed to the field by generating a comprehensive single-cell transcriptomic atlas of the retina under HP, offering a valuable resource for future research. However, HP has inherent limitations. While it facilitates the exchange of systemic factors, it does not fully capture the complexity of natural aging, which involves prolonged interactions with diverse environmental and cellular factors. Moreover, HP does not account for tissue-specific microenvironments or local regulatory mechanisms that influence aging independently of systemic circulation. Although each single-cell sample was derived from pooled retinas of three mice, our sequencing data, to some extent, lacked independent biological replicates at the single-cell level. Future studies incorporating additional biological replicates will be necessary to further validate our findings.

Detachment of parabionts is not a standard procedure in HP studies, but it has been reported in previous research (*26*, *45*), particularly when collecting certain physiological data (e.g., weight), assessing lifespan extension, and performing behavioral tests. In our study, detachment was necessary for ERG recordings because of anesthesia constraints. Proper ERG assessment requires precise anesthetic dosing, which is difficult in conjoined parabionts because of shared circulation and challenges in individual weight measurement. While detachment surgery inevitably introduces some degree of stress, whether this stress translates into meaningful physiological alterations remains uncertain. A previous study has shown that parabiosis and subsequent detachment do not affect key biological parameters, such as epigenetic age (*26*). Similarly, our results indicated that the surgical procedure itself did not affect retinal function.

Despite the up-regulation of AdipoR1 upon exposure to young circulation, serum adiponectin levels were not rescued by HP. This suggests that AdipoR1 up-regulation in Ahet mice is not solely driven by circulating adiponectin levels. Instead, it might result from broader systemic rejuvenation effects, such as improved inflammatory or metabolic states, which could enhance AdipoR1 expression and signaling capacity. This aligned with a previous study showing that receptor expression can be regulated by multiple factors beyond its ligand concentration, including local cellular conditions and other systemic mediators (*46*).

Moreover, several members of the C1q/TNF–related protein (CTRP) family have been identified as alternative ligands for AdipoR1, capable of exerting adipocytokine-like effects (*47*–*49*). In the context of retinal degeneration, studies have revealed that while both CTRP9 and adiponectin can bind AdipoR1, the knockout of either gene results in milder retinal phenotypes compared to AdipoR1 knockout

mice, with adiponectin-deficient mice exhibiting virtually no retinal abnormalities (*50*, *51*). This suggests that AdipoR1 may integrate signals from multiple ligands or function through broader, potentially ligand-independent mechanisms. In our study, this supported the idea that AdipoR1 itself may play a more pivotal role in retinal rejuvenation than any single ligand. Future studies using ligand-specific gain- or loss-of-function models to identify additional AdipoR1-binding proteins will help clarify the rejuvenation factors related to AdipoR1.

AdipoR1 has been shown to confer antiaging benefits in various systems, mitigating processes such as cellular senescence, chronic inflammation, and metabolic dysfunction (*52*–*56*). Furthermore, its activation has been linked to protection against aging-related diseases, including Alzheimer's disease, cardiovascular disorders, type 2 diabetes, and its complications, through pathways involving improved mitochondrial function, enhanced oxidative stress resistance, and metabolic reprogramming (*34*–*36*, *57*, *58*). In the context of our study, these findings shed light on the potential dual benefits of activating AdipoR1 with systemic AR treatment. Beyond directly activating the AMPK-mitochondria pathway within the retina, systemic AdipoR1 activation may enhance overall physiological conditions, indirectly supporting retinal rejuvenation by reducing circulating proaging factors and promoting metabolic homeostasis.

In the retina, AdipoR1 has been reported to play a critical role in maintaining cellular energy homeostasis and photoreceptor integrity (*51*, *59*, *60*). Our findings further elucidated AdipoR1's contribution to alleviating retinal aging and senescence and highlighted its potential as a therapeutic target for age-related retinal degeneration, expanding its known antiaging effects into the domain of retinal health.

The retina is a metabolically demanding tissue and relies on high energy turnover to sustain neural signaling, phototransduction, and synaptic transmission. This substantial energy requirement renders it particularly susceptible to bioenergetic decline with aging (*61*). AMPK, a critical regulator of cellular energy homeostasis, mitigates energy deficits by enhancing ATP production and curbing energy expenditure. However, aging diminishes AMPK activation responsiveness, potentially due to the increased activity of protein phosphatases (*62*).

Our findings reveal that HP and AR treatment effectively reverse retinal bioenergetic deficits. Transcriptomic analysis highlighted the reactivation of AMPK signaling and the enrichment of energy metabolism pathways in Ahet retinas. Functionally, AR restored mitochondrial oxidative phosphorylation, improved membrane potential, and increased ATP levels. Mitophagy flux was further enhanced, facilitating the removal of damaged mitochondria and maintaining energy homeostasis. Moreover, AdipoR1-mediated AMPK activation may extend beyond merely meeting energy demands; it could also underpin the restoration of postsynaptic dendritic extension in HCs and RBCs with retinal aging as AMPK has been reported to regulate synaptic remodeling in old age (*62*).

Mitochondrial dysfunction is one of the hallmarks of aging. Aging-related mitochondrial function deterioration arises from mtDNA mutations, disrupted proteostasis, impaired turnover, and altered dynamics (*63*). These changes not only weaken mitochondria's role as a powerhouse but also render them key contributors to tissue inflammation through elevated ROS and cytosolic mtDNA release. Recent findings have demonstrated that induction of mitophagy alleviates the cytosolic mtDNA-activated cGAS/STING inflammation and attenuates the neurological decline in the aged retina (*64*), further supporting the rejuvenation effect of AdipoR1-induced mitophagy.

Future research could build on our findings by integrating complementary models, such as human organoids and advanced in vivo systems, to validate the identified mechanisms and enhance their relevance to human biology. These approaches may help bridge the gaps in our understanding of how systemic and local factors interact in the aging process, ultimately guiding more effective therapeutic strategies.

MATERIALS AND METHODS

Experimental design

The study aimed to identify rejuvenation players capable of reversing retinal aging using HP and scRNA-seq. Initially, transcriptional profiles of young and aged retinas were analyzed to investigate aging-associated alterations across multiple retinal cell types. HP pairs and age-matched control pairs were then established to examine the effects of systemic factors and uncover key antiaging candidates. Integrative bioinformatic analysis identified AdipoR1 as a potential rejuvenating player, which was further validated through treatment with its agonist AR. The underlying mechanisms of AdipoR1-mediated retinal rejuvenation were also elucidated. Mice were randomly allocated to each group. Investigators for measurement, quantification, and data collection were blinded to the treatment of the samples to avoid human bias. In general, 3 to 12 biological replicates in each group were used in this study. The exact numbers of biological replicates in different experiments were stated in the figure legends. Sample sizes were calculated on the basis of similar types of prior studies, where $\alpha = 0.05$, $(1 - \beta) = 0.8$, and effect size ≥ 2.

Animals and drug treatment

Female mice of the C57BL/6J wild-type strain were acquired from the Guangdong Medical Laboratory Animal Center. The mice were housed in a controlled, pathogen-free environment within the animal laboratories. The experiments conducted in this study were authorized by the Institutional Animal Care and Use Committee of Zhongshan Ophthalmic Center, Sun Yat-sen University (J2024377). The mice were kept in a controlled environment with a 12-hour light/dark cycle. For the parabiosis experiment, mice were divided into the following four groups: (i) Yiso group, consisting of 6- to 8-week-old mice; (ii) Aiso group, consisting of 18- to 20-month-old mice; (iii) Yhet group consisting of young mice that received HP; (iv) Ahet group with aged mice that received HP.

For the AR experiment, mice were divided into the following three groups: (i) young group, consisting of 6- to 8-week-old mice that received standard water and food ad libitum; (ii) aged group, consisting of 18- to 20-month-old mice that received standard water and food ad libitum; (iii) AR group, consisting of 18- to 20-month-old mice that received water and tailor-made food ad libitum. AR (no. S7365, Selleck) was mixed with regular chow with a concentration of 100 mg/kg, which was an approximate average dosage of 40 mg/kg·day for each mouse. The administration of AR lasted for 2 months.

Parabiosis and detachment surgery

Parabiosis surgery was performed in a sterile facility following previously established protocols (*65*). Mice were anesthetized via intraperitoneal injection of pentobarbital sodium (10 mg/kg). The designated attachment site was shaved in a continuous line from the elbow to the knee, followed by disinfection with povidone-iodine. A

skin incision was made along the flank, extending from the proximal knee to the elbow, while ensuring that the underlying muscle tissue remained intact.

To establish a shared circulatory system, the corresponding triceps and quadriceps muscles of both mice were carefully approximated and secured using two interrupted 5-0 absorbable sutures. The skin was then aligned and closed with interrupted 5-0 nonabsorbable sutures. Postoperatively, the conjoined mice were placed in a supine position on a heated pad and monitored until full recovery. Each parabiont was housed separately in a standard cage with free access to food and water. To minimize postoperative stress and facilitate recovery, analgesics (buprenorphine, 0.1 mg/kg, subcutaneously) were administered every 12 hours for 48 hours.

Following the surgery, each pair of parabionts was housed separately for 2 months before being euthanized or detached for ERG analysis. The separation procedure was performed by reversing the initial surgical steps as previously described (*26*). Detailed postoperative care was also applied.

Cliff test

A transparent plastic box (48 cm by 48 cm by 30 cm) was set up so that it was half rested on a table (shallow side) and half hung 60 cm above the floor (deep side). The same checkered pattern was placed under both sides—on the table for the shallow part and on the floor for the deep part. Mice were placed on the shallow side near the edge and watched for 5 min. The times spent on the deep and shallow sides were recorded separately. Each mouse was subjected to one test, and the box was cleaned after every test to avoid leftover scents or contamination.

Looming visual stimulus test

An open-top box (48 cm by 48 cm by 30 cm) made of frosted plastic was used to avoid light glare. A small triangular shelter (20 cm by 12 cm) was put in one corner, and food was placed in the opposite corner to lure mice out. A screen showing a moving black circle was placed above the box. The circle started small (2° of the mouse's view), grew big (20°) in a quarter of a second, stayed big for another quarter second, and repeated 15 times with half-second breaks. A mouse was considered a responder to the stimulus if it demonstrated at least one of two behaviors: freezing or fleeing, which was recorded by the camera. Each mouse did only one test, and the box was cleaned after each test to avoid leftover scents or contamination.

Murine ERG

A Celeris D430 rodent ERG system (Diagnosys LLC, MA) was used to perform full-field ERG recording. After overnight dark adaptation, mice were anesthetized intraperitoneally using a ketamine-xylazine mixture according to the manual guide. The pupils underwent dilation using compound tropicamide eye drops for a duration of 5 min. The mice were positioned on a platform heater set to a temperature of 37°C. The corneal electrodes, along with an integrated stimulator, were applied to both eyes at the same time following the application of 1 to 2% hydroxypropyl methylcellulose as a lubricant. We chose the TOUCH/TOUCH protocol for bilateral detection, with the unstimulated side serving as a reference. Initially, scotopic ERG was conducted using ascending light intensities ranging from 0.01 to 3 cd·s/m^2. A 10-min light adaptation was conducted using a background light intensity of 30 cd·s/m^2. Photopic ERG was subsequently conducted at luminance levels of 3 and 10 cd·s/m^2. Ten sweeps were performed for each light stimulus. The pretrigger and posttrigger times were set to 50 and 300 ms, respectively, with a sampling frequency of 2000 Hz. The amplitude of the a-wave was determined by subtracting the baseline from the trough, while the b-wave amplitude was measured as the difference between the trough of the a-wave and the peak of the highest curve.

IF staining

The mice were anesthetized using a 1% isoflurane solution, followed by transcardial perfusion with a 0.9% saline solution and 4% paraformaldehyde solution. The entire eyeballs were collected and afterward immersed in a 4% paraformaldehyde solution at a temperature of 4°C for a duration of 2 hours. The tissue was subjected to dehydration in a 30% sucrose solution overnight. The tissues were embedded in an optimum cutting temperature compound (SAKURA, Japan) and subsequently cryosectioned at 14 μm in thickness. The sections were subjected to permeabilization using a solution containing 0.3% Triton X-100. Subsequently, they were blocked using a solution containing 2% bovine serum albumin and 10% normal donkey serum. The samples were then subjected to incubation with primary antibodies at a temperature of 4°C for the duration of the overnight period. Following this, a secondary antibody that is compatible with the species was applied to the samples and allowed to incubate for a period of 2 hours at room temperature. For cell IF staining, cells were cultured on Nunc Lab-Tek chamber slides (Thermo Fisher Scientific), fixed with 4% paraformaldehyde, and permeabilized with 0.1% Triton X-100. After blocking with 5% bovine serum albumin, samples were incubated with primary antibodies overnight at 4°C, followed by fluorescently labeled secondary antibodies at room temperature. Nuclei were counterstained with DAPI (4′,6-diamidino-2-phenylindole; Bioss). The primary antibody specifications were provided as follows: anti-GFAP (no. ab194324, Abcam) at 1:500, anti-Iba1 (no. 019-19741, Wako) at 1:500, anti-PKCα (protein kinase Cα; no. ab32376, Abcam) at 1:100, calbindin (no. 13176T, Cell Signaling Technology) at 1:100, MitoTracker Green (no. M46750, Invitrogen) at 1:1000, and LC3II (no. ab48394, Abcam). Images were captured by a Nikon confocal microscope (C2+, Nikon, Japan). The images were further processed using ImageJ software (https://imagej.net/ij/).

FC analysis

To analyze the retina, we extracted the mouse retina from various groups and isolated the cell suspensions by grinding the organs using nylon mesh. Cell lines were acquired from various groups using enzyme digestion for analysis purposes. Dead cells were excluded using a live/dead dye (no. 423105, BioLegend). Subsequently, the cells were stained using the following surface antibodies: CD11b BV605 (no. 101237, BioLegend) and Adipor1 (no. MA5-32249, Invitrogen). To stain intracellular markers, cells were stimulated with specific concentrations of phorbol myristate acetate, ionomycin, and brefeldin A (Sigma-Aldrich) at 37°C for 5 hours. Subsequently, the cells were fixed and permeabilized. The cells were stained with the following antibodies: IL-6 PE (no. 504504, BioLegend), TNF-α BV421 (no. 506327, BioLegend), IL-1β PE-Cy7 (no. 25-7114-80, Invitrogen, Carlsbad, CA), and p21 Alexa Fluor 647 (no. ab237265, Abcam) for mouse retina and primary microglia. For ARPE-19, the cells were stained with p53 PE (no. 645806, BioLegend) and p21 Alexa Fluor 488 (no. ab282187, Abcam). For p16 staining, cells were first stained with surface antibodies. They were then fixed, permeabilized, and stained with the p16 antibody (no. ab211542, Abcam).

Last, the cells were stained with an Alexa Fluor 647–labeled antibody (no. 4414S, Cell Signaling Technology, Danvers, US). Cells were stained with the senescence assay (no. ab228562, Abcam) for β-GAL staining, following the manufacturer's instructions. To assess mitochondrial ROS production and membrane potential, MSR Mitochondrial Superoxide Indicator (no. M36008, Invitrogen) and TMRM Reagent (no. I34361, Invitrogen) were used according to the manufacturer's instructions. To evaluate the mitophagy flux, retinal cells were in vitro treated with CCCP (10 μM, no. C2759, Sigma-Aldrich) for 6 hours to induce mitophagy followed by HCQ (30 μM, no. E4824, Selleck) for 3 hours before FC analysis to block lysosomal degradation. Then, cells were stained with MitoTracker Green (no. M46750, Invitrogen). A flow cytometer (BD LSRFortessa, US) was used for analysis, and the resulting data were analyzed using FlowJo software (version 10.0.7, Tree Star).

Western blot

Retinal protein was isolated using a radioimmunoprecipitation assay lysis solution (Beyotime, China) and separated on 12% (w/v) gradient polyacrylamide gels according to a standard protocol. The expression levels of target proteins were standardized to β-actin or GAPDH (glyceraldehyde-3-phosphate dehydrogenase), which was obtained from the same sample and set as a reference value of 1.0. The quantification of protein expression was performed using ImageJ Software, available at https://imagej.net/ij/. The primary antibodies and their dilutions were shown as follows: anti-AdipoR1 SC69-04 (no. NBP2-67631, NOVUS) at 1:1000, anti-AMPKα (no. 2532S, CST) at 1:1000, anti–Phospho-AMPKα (Thr^{172}) (no. 2531S, CST) at 1:1000, anti–β-actin (no. 4970S, CST) at 1:2500, and anti-GAPDH (no. 60004-1-1G, Proteintech) at 1:2500.

OCR examination

OCR of 661W cells was assessed using a Seahorse XF24 Analyzer. Data were normalized to cell numbers, and metabolic parameters were automatically calculated by Seahorse software. Oligomycin, FCCP (carbonyl cyanide ptrifluoromethoxyphenylhydrazone), and rotenone and antimycin were sequentially injected as specified.

ATP production analysis

Mitochondrial ATP levels were evaluated with the ATP Determination Kit (A22066, Invitrogen) following the manufacturer's instructions.

Enzyme-linked immunosorbent assay (ELISA)

The sera from mice were determined using the mouse adiponectin ELISA Kit (no. SEA605Mu, USCN, Wuhan, Hubei Province, China) according to the manual guide.

Isolation and culture of primary microglia

Primary retinal microglia were isolated and cultured from wild-type Sprague-Dawley rat pups at postnatal day 3. Following euthanasia by decapitation in accordance with institutional ethical guidelines, eyes were enucleated using sterile fine scissors and forceps, and retinas were dissected in ice-cold calcium- and magnesium-free Hanks' balanced salt solution under a stereomicroscope. Typically, each pair of retinas was placed in a 2-ml Eppendorf tube containing Dulbecco's modified Eagle's medium (DMEM) supplemented with 20% fetal bovine serum (FBS) and kept on ice until processing. The retinas were mechanically dissociated by pipetting up and down with a 1-ml pipette for 5 min. The resulting cell suspension was filtered through a 70-μm cell strainer to remove debris and centrifuged at 1000 rpm for 5 min at 4°C. Cells were plated onto poly-L-lysine–coated T-75 flasks or culture dishes and maintained in a humidified incubator at 37°C with 5% CO_2. After 7 to 10 days, when a confluent mixed glial culture was established, microglia were selectively detached by gentle shaking (500 rpm for 1 hour at 37°C) and collected from the supernatant. The isolated microglia were then centrifuged at 1000 rpm for 5 min, resuspended in fresh DMEM (10% FBS), and seeded into dishes for downstream experiments. Microglial identity was confirmed by FC using a CD11b marker.

Cell culture and treatment

The 661W photoreceptor cell line and ARPE-19 were obtained from the American Type Culture Collection in the United States. All cell lines were cultured under standard conditions of 37°C, 5% CO_2, and normoxia. 661W and ARPE-19 cells were maintained in DMEM/F12 medium (Gibco). The medium was supplemented with 10% FBS to complete the growth medium. To induce senescence, 661W and ARPE-19 cells were treated with 200 μM H_2O_2 (no. 7722-84-1, Sigma-Aldrich) in complete medium for 4 hours daily over four consecutive days. Throughout the remaining period, the cells were maintained in complete medium. After the 4-day treatment, cells were cultured in complete medium for an additional 2 days before being harvested. In the case of primary microglia, the stimulant was changed from H_2O_2 to LPS (10 ng/ml; no. 297-473-0, Sigma-Aldrich), as repeated LPS stimulation has been shown to effectively induce cellular senescence in microglia (*5*, *37*). The treatment schedule for primary microglia mirrored that used for 661W and ARPE-19 cells. The AR-treated groups were exposed to the agent at a concentration of 50 μM for a duration of 24 hours.

scRNA-seq analysis

For each scRNA-seq experiment, three mice were used per group. The construction of barcoded libraries was accomplished using the 10x Genomics kit. The sequencing data were processed using the CellRanger (version 7.0.0) count pipeline and integrated using the CellRanger aggr command. The analysis was conducted in R using the Seurat package (version 4.1.1) with default parameters, unless stated otherwise. Cells meeting quality control criteria were selected on the basis of the following criteria: having more than 200 and fewer than 4000 genes, as well as less than 20% mitochondrial genes. These cells were subsequently used for further analysis. The R package harmony (version 0.1.0) was used to mitigate the batch effect observed in various sequencing samples. Differential expression analysis was conducted using the "FindMarkers" function within the Seurat package. DEGs were identified on the basis of the criteria of $|\log_2(\text{fold change})| > 0.25$ and adjusted P value <0.05, with P values corrected for multiple testing using the Benjamini-Hochberg method.

Gene functional analysis

DEGs were functionally annotated using GO analysis in Metascape (*66*). The enrichment P values are adjusted using the Benjamini-Hochberg procedure to control the false discovery rate. All reported pathway analyses in our study used adjusted P values. The R package ggplot2 (version 3.3.6) showcased a selection of representative GO terms or pathways.

TF analysis

TF analysis was conducted using the pySCENIC workflow with default parameters based on the mm10 database.

Integrative analysis with public datasets

Gene sets related to aging-related retinal diseases were obtained from the database DisGeNET (www.disgenet.org/). Gene sets were generated by filtering with "diseaseName" ("age-related macular degeneration," "diabetic retinopathy," and "glaucoma"). Age-related genes were acquired from public databases including Aging Atlas, GenAge, and CellAge.

Gene set score analysis

Genes associated with the SASP, retina homeostasis, and AMPK signaling pathways were obtained from MSigDb (www.gsea-msigdb.org/gsea/index.jsp) and Harmonizome (https://maayanlab.cloud/Harmonizome/). The gene set scores were computed using the "AddModuleScore" function from the Seurat package in R.

Statistical analysis

The data were reported as the means ± SD, unless otherwise specified. The two-tailed Student's *t* test was used to compare two groups. A one-way analysis of variance (ANOVA) with a Bonferroni post test was used to statistically analyze variables across multiple groups. For gene set score analysis, statistical analysis was performed using two-sided Wilcoxon rank-sum tests with Benjamini-Hochberg correction to adjust for multiple testing. A *P* value less than 0.05 was deemed to be statistically significant. The figure legends provided information on both the sample sizes and *P* values. Statistical analysis was conducted using GraphPad Prism 9 (GraphPad Software, United States) and R software (version 4.1.3, R Foundation for Statistical Computing, Austria).

Supplementary Materials

This PDF file includes:
Figs. S1 to S7
Tables S1 and S2

REFERENCES AND NOTES

1. D. Muñoz-Espín, M. Serrano, Cellular senescence: From physiology to pathology. *Nat. Rev. Mol. Cell Biol.* **15**, 482–496 (2014).
2. A. P. Marques, J. Ramke, J. Cairns, T. Butt, J. H. Zhang, I. Jones, M. Jovic, A. Nandakumar, H. Faal, H. Taylor, A. Bastawrous, T. Braithwaite, S. Resnikoff, P. T. Khaw, R. Bourne, I. Gordon, K. Frick, M. J. Burton, The economics of vision impairment and its leading causes: A systematic review. *EClinicalMedicine* **46**, 101354 (2022).
3. S. R. Flaxman, R. R. A. Bourne, S. Resnikoff, P. Ackland, T. Braithwaite, M. V. Cicinelli, A. Das, J. B. Jonas, J. Keeffe, J. H. Kempen, J. Leasher, H. Limburg, K. Naidoo, K. Pesudovs, A. Silvester, G. A. Stevens, N. Tahhan, T. Y. Wong, H. R. Taylor, Vision Loss Expert Group of the Global Burden of Disease Study, Global causes of blindness and distance vision impairment 1990-2020: A systematic review and meta-analysis. *Lancet Glob. Health* **5**, e1221–e1234 (2017).
4. M. Campbell, A. T. H. Nguyen, A.-S. Kiang, L. C. S. Tam, O. L. Gobbo, C. Kerskens, S. Ni Dhubhghaill, M. M. Humphries, G.-J. Farrar, P. F. Kenna, P. Humphries, An experimental platform for systemic drug delivery to the retina. *Proc. Natl. Acad. Sci. U.S.A.* **106**, 17817–17822 (2009).
5. Y. Liu, X. Liu, X. Chen, Z. Yang, J. Chen, W. Zhu, Y. Li, Y. Wen, C. Deng, C. Gu, J. Lv, R. Ju, Y. Zhuo, W. Su, Senolytic and senomorphic agent procyanidin C1 alleviates structural and functional decline in the aged retina. *Proc. Natl. Acad. Sci. U.S.A.* **121**, e2311028121 (2024).
6. B. D. Wagner, J. L. Patnaik, A. G. Palestine, A. A. Frazer-Abel, R. Baldermann, V. M. Holers, M. T. Mathias, N. Mandava, A. M. Lynch, Association of systemic inflammatory factors with progression to advanced age-related macular degeneration. *Ophthalmic Epidemiol.* **29**, 139–148 (2022).
7. H. Xu, M. Chen, J. V. Forrester, Para-inflammation in the aging retina. *Prog. Retin. Eye Res.* **28**, 348–368 (2009).
8. H. W. van Dijk, F. D. Verbraak, P. H. B. Kok, M. Stehouwer, M. K. Garvin, M. Sonka, J. H. DeVries, R. O. Schlingemann, M. D. Abràmoff, Early neurodegeneration in the retina of type 2 diabetic patients. *Invest. Ophthalmol. Vis. Sci.* **53**, 2715–2719 (2012).
9. H. Zhang, Y. Mo, The gut-retina axis: A new perspective in the prevention and treatment of diabetic retinopathy. *Front. Endocrinol.* **14**, 1205846 (2023).
10. C. Y. Cheung, W. T. Tay, P. Mitchell, J. J. Wang, W. Hsu, M. L. Lee, Q. P. Lau, A. L. Zhu, R. Klein, S. M. Saw, T. Y. Wong, Quantitative and qualitative retinal microvascular characteristics and blood pressure. *J. Hypertens.* **29**, 1380–1391 (2011).
11. S. A. Villeda, K. E. Plambeck, J. Middeldorp, J. M. Castellano, K. I. Mosher, J. Luo, L. K. Smith, G. Bieri, K. Lin, D. Berdnik, R. Wabl, J. Udeochu, E. G. Wheatley, B. Zou, D. A. Simmons, X. S. Xie, F. M. Longo, T. Wyss-Coray, Young blood reverses age-related impairments in cognitive function and synaptic plasticity in mice. *Nat. Med.* **20**, 659–663 (2014).
12. S. Ma, S. Wang, Y. Ye, J. Ren, R. Chen, W. Li, J. Li, L. Zhao, Q. Zhao, G. Sun, Y. Jing, Y. Zuo, M. Xiong, Y. Yang, Q. Wang, J. Lei, S. Sun, X. Long, M. Song, S. Yu, P. Chan, J. Wang, Q. Zhou, J. C. I. Belmonte, J. Qu, W. Zhang, G.-H. Liu, Heterochronic parabiosis induces stem cell revitalization and systemic rejuvenation across aged tissues. *Cell Stem Cell* **29**, 990–1005.e10 (2022).
13. M. J. Yousefzadeh, J. E. Wilkinson, B. Hughes, N. Gadela, W. C. Ladiges, N. Vo, L. J. Niedernhofer, D. M. Huffman, P. D. Robbins, Heterochronic parabiosis regulates the extent of cellular senescence in multiple tissues. *GeroScience* **42**, 951–961 (2020).
14. M. Ximerakis, K. M. Holton, R. M. Giadone, C. Ozek, M. Saxena, S. Santiago, X. Adiconis, D. Dionne, L. Nguyen, K. M. Shah, J. M. Goldstein, C. Gasperini, I. A. Gampierakis, S. L. Lipnick, S. K. Simmons, S. M. Buchanan, A. J. Wagers, A. Regev, J. Z. Levin, L. L. Rubin, Heterochronic parabiosis reprograms the mouse brain transcriptome by shifting aging signatures in multiple cell types. *Nat. Aging* **3**, 327–345 (2023).
15. R. Pálovics, A. Keller, N. Schaum, W. Tan, T. Fehlmann, M. Borja, F. Kern, L. Bonanno, K. Calcuttawala, J. Webber, A. McGeever, Tabula Muris Consortium, J. Luo, A. O. Pisco, J. Karkanias, N. F. Neff, S. Darmanis, S. R. Quake, T. Wyss-Coray, Molecular hallmarks of heterochronic parabiosis at single-cell resolution. *Nature* **603**, 309–314 (2022).
16. O. H. Jeon, M. Mehdipour, T.-H. Gil, M. Kang, N. W. Aguirre, Z. R. Robinson, C. Kato, J. Etienne, H. G. Lee, F. Alimirah, V. Walavalkar, P.-Y. Desprez, M. J. Conboy, J. Campisi, I. M. Conboy, Systemic induction of senescence in young mice after single heterochronic blood exchange. *Nat. Metab.* **4**, 995–1006 (2022).
17. I. M. Conboy, M. J. Conboy, A. J. Wagers, E. R. Girma, I. L. Weissman, T. A. Rando, Rejuvenation of aged progenitor cells by exposure to a young systemic environment. *Nature* **433**, 760–764 (2005).
18. J. M. Castellano, E. D. Kirby, T. Wyss-Coray, Blood-borne revitalization of the aged brain. *JAMA Neurol.* **72**, 1191–1194 (2015).
19. G. Bieri, A. B. Schroer, S. A. Villeda, Blood-to-brain communication in aging and rejuvenation. *Nat. Neurosci.* **26**, 379–393 (2023).
20. L. Katsimpardi, N. K. Litterman, P. A. Schein, C. M. Miller, F. S. Loffredo, G. R. Wojtkiewicz, J. W. Chen, R. T. Lee, A. J. Wagers, L. L. Rubin, Vascular and neurogenic rejuvenation of the aging mouse brain by young systemic factors. *Science* **344**, 630–634 (2014).
21. S. Wang, Y. Zheng, Q. Li, X. He, R. Ren, W. Zhang, M. Song, H. Hu, F. Liu, G. Sun, S. Sun, Z. Liu, Y. Yu, P. Chan, G.-G. Zhao, Q. Zhou, G.-H. Liu, F. Tang, J. Qu, Deciphering primate retinal aging at single-cell resolution. *Protein Cell* **12**, 889–898 (2021).
22. A. Podleśny-Drabiniok, G. Novikova, Y. Liu, J. Dunst, R. Temizer, C. Giannarelli, S. Marro, T. Kreslavsky, E. Marcora, A. M. Goate, BHLHE40/41 regulate microglia and peripheral macrophage responses associated with Alzheimer's disease and other disorders of lipid-rich tissues. *Nat. Commun.* **15**, 2058 (2024).
23. A. K. Vijaya, S. Kuras, E. Šimoliūnas, J. Mingaila, K. Makovskytė, R. Buišas, E. B.-M. Daliri, R. Meškys, D. Baltriukienė, A. Burokas, Prebiotics mitigate the detrimental effects of high-fat diet on memory, anxiety and microglia functionality in ageing mice. *Brain Behav. Immun.* **122**, 167–184 (2024).
24. S. Zhou, T. Li, W. Zhang, J. Wu, H. Hong, W. Quan, X. Qiao, C. Cui, C. Qiao, W. Zhao, Y. Shen, The cGAS-STING-interferon regulatory factor 7 pathway regulates neuroinflammation in Parkinson's disease. *Neural Regen. Res.* **20**, 2361–2372 (2025).
25. M. Hata, M. Hata, E. M. Andriessen, R. Juneau, F. Pilon, S. Crespo-Garcia, R. Diaz-Marin, V. Guber, F. Binet, F. Fournier, M. Buscarlet, C. Grou, V. Calderon, E. Heckel, H. J. Melichar, J.-S. Joyal, A. M. Wilson, P. Sapieha, Early-life peripheral infections reprogram retinal microglia and aggravate neovascular age-related macular degeneration in later life. *J. Clin. Invest.* **133**, e159757 (2023).
26. B. Zhang, D. E. Lee, A. Trapp, A. Tyshkovskiy, A. T. Lu, A. Bareja, C. Kerepesi, L. K. McKay, A. V. Shindyapina, S. E. Dmitriev, G. S. Baht, S. Horvath, V. N. Gladyshev, J. P. White, Multi-omic rejuvenation and lifespan extension on exposure to youthful circulation. *Nat. Aging* **3**, 948–964 (2023).
27. R. Li, Y. Liang, B. Lin, Accumulation of systematic TPM1 mediates inflammation and neuronal remodeling by phosphorylating PKA and regulating the FABP5/NF-κB signaling pathway in the retina of aged mice. *Aging Cell* **21**, e13566 (2022).
28. M. Mehdipour, T. Mehdipour, C. M. Skinner, N. Wong, C. Liu, C.-C. Chen, O. H. Jeon, Y. Zuo, M. J. Conboy, I. M. Conboy, Plasma dilution improves cognition and attenuates neuroinflammation in old mice. *GeroScience* **43**, 1–18 (2021).
29. J. M. van Deursen, The role of senescent cells in ageing. *Nature* **509**, 439–446 (2014).
30. D. Saul, R. L. Kosinsky, E. J. Atkinson, M. L. Doolittle, X. Zhang, N. K. LeBrasseur, R. J. Pignolo, P. D. Robbins, L. J. Niedernhofer, Y. Ikeno, D. Jurk, J. F. Passos, L. J. Hickson,

A. Xue, D. G. Monroe, T. Tchkonia, J. L. Kirkland, J. N. Farr, S. Khosla, A new gene set identifies senescent cells and predicts senescence-associated pathways across tissues. *Nat. Commun.* **13**, 4827 (2022).
31. M. Nebbioso, A. Lambiase, M. Armentano, G. Tucciarone, V. Bonfiglio, R. Plateroti, L. Alisi, The complex relationship between diabetic retinopathy and high-mobility group box: A review of molecular pathways and therapeutic strategies. *Antioxidants* **9**, 666 (2020).
32. J. Peng, J. Jin, W. Su, W. Shao, W. Li, Z. Li, H. Yu, Y. Zheng, L. Zhong, High-mobility group box 1 inhibitor BoxA alleviates neuroinflammation-induced retinal ganglion cell damage in traumatic optic neuropathy. *Int. J. Mol. Sci.* **23**, 6715 (2022).
33. M. Iwabu, M. Okada-Iwabu, T. Yamauchi, T. Kadowaki, Adiponectin/adiponectin receptor in disease and aging. *NPJ Aging Mech. Dis.* **1**, 15013 (2015).
34. R. C.-L. Ng, O.-Y. Cheng, M. Jian, J. S.-C. Kwan, P. W.-L. Ho, K. K.-Y. Cheng, P. K. K. Yeung, L. L. Zhou, R. L.-C. Hoo, S. K. Chung, A. Xu, K. S.-L. Lam, K. H. Chan, Chronic adiponectin deficiency leads to Alzheimer's disease-like cognitive impairments and pathologies through AMPK inactivation and cerebral insulin resistance in aged mice. *Mol. Neurodegener.* **11**, 71 (2016).
35. M. Iwabu, T. Yamauchi, M. Okada-Iwabu, K. Sato, T. Nakagawa, M. Funata, M. Yamaguchi, S. Namiki, R. Nakayama, M. Tabata, H. Ogata, N. Kubota, I. Takamoto, Y. K. Hayashi, N. Yamauchi, H. Waki, M. Fukayama, I. Nishino, K. Tokuyama, K. Ueki, Y. Oike, S. Ishii, K. Hirose, T. Shimizu, K. Touhara, T. Kadowaki, Adiponectin and AdipoR1 regulate PGC-1α and mitochondria by Ca2+ and AMPK/SIRT1. *Nature* **464**, 1313–1319 (2010).
36. M. Jian, J. S.-C. Kwan, M. Bunting, R. C.-L. Ng, K. H. Chan, Adiponectin suppresses amyloid-β oligomer (AβO)-induced inflammatory response of microglia via AdipoR1-AMPK-NF-κB signaling pathway. *J. Neuroinflammation* **16**, 110 (2019).
37. H.-M. Yu, Y.-M. Zhao, X.-G. Luo, Y. Feng, Y. Ren, H. Shang, Z.-Y. He, X.-M. Luo, S.-D. Chen, X.-Y. Wang, Repeated lipopolysaccharide stimulation induces cellular senescence in BV2 cells. *Neuroimmunomodulation* **19**, 131–136 (2012).
38. M. Mauro-Lizcano, L. Esteban-Martínez, E. Seco, A. Serrano-Puebla, L. Garcia-Ledo, C. Figueiredo-Pereira, H. L. A. Vieira, P. Boya, New method to assess mitophagy flux by flow cytometry. *Autophagy* **11**, 833–843 (2015).
39. A. Deczkowska, H. Keren-Shaul, A. Weiner, M. Colonna, M. Schwartz, I. Amit, Disease-associated microglia: A universal immune sensor of neurodegeneration. *Cell* **173**, 1073–1081 (2018).
40. H. Keren-Shaul, A. Spinrad, A. Weiner, O. Matcovitch-Natan, R. Dvir-Szternfeld, T. K. Ulland, E. David, K. Baruch, D. Lara-Astaiso, B. Toth, S. Itzkovitz, M. Colonna, M. Schwartz, I. Amit, A unique microglia type associated with restricting development of Alzheimer's disease. *Cell* **169**, 1276–1290.e17 (2017).
41. T. R. Hammond, C. Dufort, L. Dissing-Olesen, S. Giera, A. Young, A. Wysoker, A. J. Walker, F. Gergits, M. Segel, J. Nemesh, S. E. Marsh, A. Saunders, E. Macosko, F. Ginhoux, J. Chen, R. J. M. Franklin, X. Piao, S. A. McCarroll, B. Stevens, Single-cell RNA sequencing of microglia throughout the mouse lifespan and in the injured brain reveals complex cell-state changes. *Immunity* **50**, 253–271.e6 (2019).
42. D. H. Anderson, R. F. Mullins, G. S. Hageman, L. V. Johnson, A role for local inflammation in the formation of drusen in the aging eye. *Am. J. Ophthalmol.* **134**, 411–431 (2002).
43. A. Ashok, N. Singh, S. Chaudhary, V. Bellamkonda, A. E. Kritikos, A. S. Wise, N. Rana, D. McDonald, R. Ayyagari, Retinal degeneration and Alzheimer's disease: An evolving link. *Int. J. Mol. Sci.* **21**, 7290 (2020).
44. K. Kaarniranta, J. Blasiak, P. Liton, M. Boulton, D. J. Klionsky, D. Sinha, Autophagy in age-related macular degeneration. *Autophagy* **19**, 388–400 (2023).
45. T. T. Ho, P. V. Dellorusso, E. V. Verovskaya, S. T. Bakker, J. Flach, L. K. Smith, P. B. Ventura, O. M. Lansinger, A. Hérault, S. Y. Zhang, Y.-A. Kang, C. A. Mitchell, S. A. Villeda, E. Passegué, Aged hematopoietic stem cells are refractory to bloodborne systemic rejuvenation interventions. *J. Exp. Med.* **218**, e20210223 (2021).
46. B. Desbuquois, E. Tozzo, M. Collinet, S. Lopez, S. Bortoli, M. Amessou, Regulation of insulin receptor expression and its gene. *Ann. Endocrinol. (Paris)* **54**, 373–384 (1993).
47. G. W. Wong, S. A. Krawczyk, C. Kitidis-Mitrokostas, G. Ge, E. Spooner, C. Hug, R. Gimeno, H. F. Lodish, Identification and characterization of CTRP9, a novel secreted glycoprotein, from adipose tissue that reduces serum glucose in mice and forms heterotrimers with adiponectin. *FASEB J.* **23**, 241–258 (2009).
48. N. Ouchi, K. Walsh, Cardiovascular and metabolic regulation by the adiponectin/C1q/tumor necrosis factor–related protein family of proteins. *Circulation* **125**, 3066–3068 (2012).
49. Q. Zheng, Y. Yuan, W. Yi, W. B. Lau, Y. Wang, X. Wang, Y. Sun, B. L. Lopez, T. A. Christopher, J. M. Peterson, G. W. Wong, S. Yu, D. Yi, X.-L. Ma, C1q/TNF-related proteins, a family of novel adipokines, induce vascular relaxation through the adiponectin receptor-1/AMPK/eNOS/nitric oxide signaling pathway. *Arterioscler. Thromb. Vasc. Biol.* **31**, 2616–2623 (2011).
50. D. Inooka, Y. Omori, N. Ouchi, K. Ohashi, Y. Kawakami, Y. Koyanagi, C. Koike, H. Terasaki, K. M. Nishiguchi, S. Ueno, Ablation of Ctrp9, ligand of AdipoR1, and lower number of cone photoreceptors in mouse retina. *Invest. Ophthalmol. Vis. Sci.* **63**, 14 (2022).
51. D. S. Rice, J. M. Calandria, W. C. Gordon, B. Jun, Y. Zhou, C. M. Gelfman, S. Li, M. Jin, E. J. Knott, B. Chang, A. Abuin, T. Issa, D. Potter, K. A. Platt, N. G. Bazan, Adiponectin receptor 1 conserves docosahexaenoic acid and promotes photoreceptor cell survival. *Nat. Commun.* **6**, 6228 (2015).
52. M. Okada-Iwabu, M. Iwabu, T. Yamauchi, T. Kadowaki, Drug development research for novel adiponectin receptor-targeted antidiabetic drugs contributing to healthy longevity. *Diabetol. Int.* **10**, 237–244 (2019).
53. R. Liu, J. Meng, D. Lou, Adiponectin inhibits D-gal-induced cardiomyocyte senescence via AdipoR1/APPL1. *Mol. Med. Rep.* **24**, 719 (2021).
54. P. Balasubramanian, A. E. Schaar, G. E. Gustafson, A. B. Smith, P. R. Howell, A. Greenman, S. Baum, R. J. Colman, D. W. Lamming, G. M. Diffee, R. M. Anderson, Adiponectin receptor agonist AdipoRon improves skeletal muscle function in aged mice. *eLife* **11**, e71282 (2022).
55. J. Sun, X. Liu, C. Shen, W. Zhang, Y. Niu, Adiponectin receptor agonist AdipoRon blocks skin inflamm-ageing by regulating mitochondrial dynamics. *Cell Prolif.* **54**, e13155 (2021).
56. M. Karamian, M. Moossavi, M. Hemmati, From diabetes to renal aging: The therapeutic potential of adiponectin. *J. Physiol. Biochem.* **77**, 205–214 (2021).
57. S. R. Choi, J. H. Lim, M. Y. Kim, E. N. Kim, Y. Kim, B. S. Choi, Y.-S. Kim, H. W. Kim, K.-M. Lim, M. J. Kim, C. W. Park, Adiponectin receptor agonist AdipoRon decreased ceramide, and lipotoxicity, and ameliorated diabetic nephropathy. *Metabolism* **85**, 348–360 (2018).
58. Y. Wang, Y. Liu, J. Fang, X. Xing, H. Wang, X. Shi, X. Liu, T. Niu, K. Liu, Small-molecule agonist AdipoRon alleviates diabetic retinopathy through the AdipoR1/AMPK/EGR4 pathway. *J. Transl. Med.* **22**, 2 (2024).
59. H. Osada, E. Toda, K. Homma, N. A. Guzman, N. Nagai, M. Ogawa, K. Negishi, M. Arita, K. Tsubota, Y. Ozawa, ADIPOR1 deficiency-induced suppression of retinal ELOVL2 and docosahexaenoic acid levels during photoreceptor degeneration and visual loss. *Cell Death Dis.* **12**, 458 (2021).
60. D. Lewandowski, A. T. Foik, R. Smidak, E. H. Choi, J. Zhang, T. Hoang, A. Tworak, S. Suh, H. Leinonen, Z. Dong, A. F. M. Pinto, E. Tom, J. Luu, J. Lee, X. Ma, E. Bieberich, S. Blackshaw, A. Saghatelian, D. C. Lyon, D. Skowronska-Krawczyk, M. Tabaka, K. Palczewski, Inhibition of ceramide accumulation in $AdipoR1^{-/-}$ mice increases photoreceptor survival and improves vision. *JCI Insight* **7**, e156301 (2022).
61. L. Campello, N. Singh, J. Advani, A. K. Mondal, X. Corso-Díaz, A. Swaroop, Aging of the retina: Molecular and metabolic turbulences and potential interventions. *Annu. Rev. Vis. Sci.* **7**, 633–664 (2021).
62. A. Salminen, K. Kaarniranta, AMP-activated protein kinase (AMPK) controls the aging process via an integrated signaling network. *Ageing Res. Rev.* **11**, 230–241 (2012).
63. C. López-Otín, M. A. Blasco, L. Partridge, M. Serrano, G. Kroemer, Hallmarks of aging: An expanding universe. *Cell* **186**, 243–278 (2023).
64. J. I. Jiménez-Loygorri, B. Villarejo-Zori, Á. Viedma-Poyatos, J. Zapata-Muñoz, R. Benítez-Fernández, M. D. Frutos-Lisón, F. A. Tomás-Barberán, J. C. Espín, E. Area-Gómez, A. Gomez-Duran, P. Boya, Mitophagy curtails cytosolic mtDNA-dependent activation of cGAS/STING inflammation during aging. *Nat. Commun.* **15**, 830 (2024).
65. P. Kamran, K.-I. Sereti, P. Zhao, S. R. Ali, I. L. Weissman, R. Ardehali, Parabiosis in mice: A detailed protocol. *J. Vis. Exp.*, 50556 (2013).
66. Y. Zhou, B. Zhou, L. Pache, M. Chang, A. H. Khodabakhshi, O. Tanaseichuk, C. Benner, S. K. Chanda, Metascape provides a biologist-oriented resource for the analysis of systems-level datasets. *Nat. Commun.* **10**, 1523 (2019).

Acknowledgments
Funding: This work was supported by the following: National Natural Science Foundation of China (824B2040 to Y.L.); National Key Research and Development Project of China (2020YFA0112701 to Y.Z.); Science and Technology Program of Guangzhou, China (202206080005 to Y.Z.); and China National Postdoctoral Program for Innovative Talents (BX20240440 to X.L.). **Author contributions:** Conceptualization: Y.L., Y.Z., and W.S. Formal analysis: Y.L. and X.L. Methodology: Y.L., X.L., Q.Z., C.G., and C.Z. Investigation: Y.L., X.L., J.L., Q.Z., and Z.Y. Resources: Z.Y. Validation: J.L., Z.Y., X.C., and C.G. Visualization: Y.L., X.L., and Q.Z. Supervision: Y.Z. and W.S. Writing—original draft: Y.L., J.L., and X.C. Writing—review and editing: X.L., Y.Z., and W.S. Project administration: Y.Z. and W.S. Funding acquisition: Y.L., X.L., and Y.Z. **Competing interests:** The authors declare that they have no competing interests. **Data and materials availability:** All data needed to evaluate the conclusions in the paper are present in the paper and/or the Supplementary Materials. The scRNA-seq data have been deposited in the publicly accessible Genome Sequence Archive at the BIG Data Center, Beijing Institute of Genomics (accession number: PRJCA030000). The data analysis pipeline in our study was described on the 10x Genomics (www.10xgenomics.com/) and the Seurat official website (https://github.com/satijalab/seurat).

Submitted 1 January 2025
Accepted 10 June 2025
Published 16 July 2025
10.1126/sciadv.adv6642
