# Supplementary material for: Heterochronic parabiosis uncovers AdipoR1 as a critical player in retinal rejuvenation
Source: Sci Adv. 2025 Jul 16;11(29):eadv6642. doi: 10.1126/sciadv.adv6642 (PMC12266120; doi:10.1126/sciadv.adv6642)
Supplement: Supplementary file 2 — Figs. S1 to S7 Tables S1 and S2 [file sciadv.adv6642_sm.pdf]

Science Advances
AAAS

Supplementary Materials for

Heterochronic parabiosis uncovers AdipoR1 as a critical player in retinal rejuvenation

Yidan Liu *et al.*

Corresponding author: Wenru Su, suwenru@sjtu.edu.cn; Yehong Zhuo, zhuoyh@mail.sysu.edu.cn

Sci. Adv. **11**, eadv6642 (2025)
DOI: 10.1126/sciadv.adv6642

This PDF file includes:

Figs. S1 to S7
Tables S1 and S2

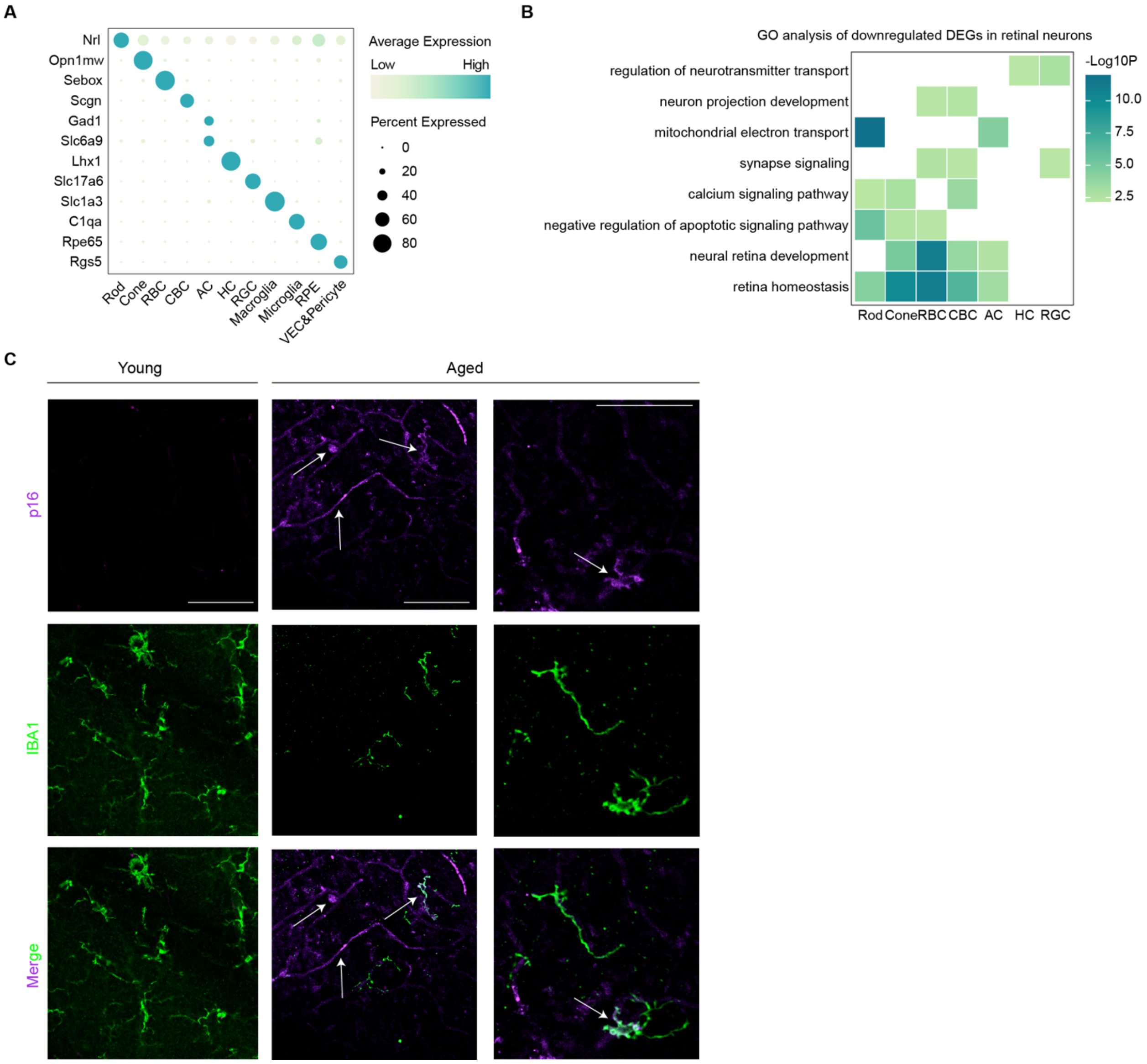

Fig. S1.

Aging altered gene expression profiles across various retinal cell types. (A) Dot plot showing the expression of cell-type-specific markers across retinal cell types. (B) Heatmap showing GO analysis of downregulated DEGs in aged retinal neurons. (C) Representative immunostaining of retinal flat mounts showing p16 (purple) and the microglial marker Iba1 (green) in young (left panel) and aged (middle and right panel) retinas. Arrows indicated the senescent cells. Scale bar: 50 μm.

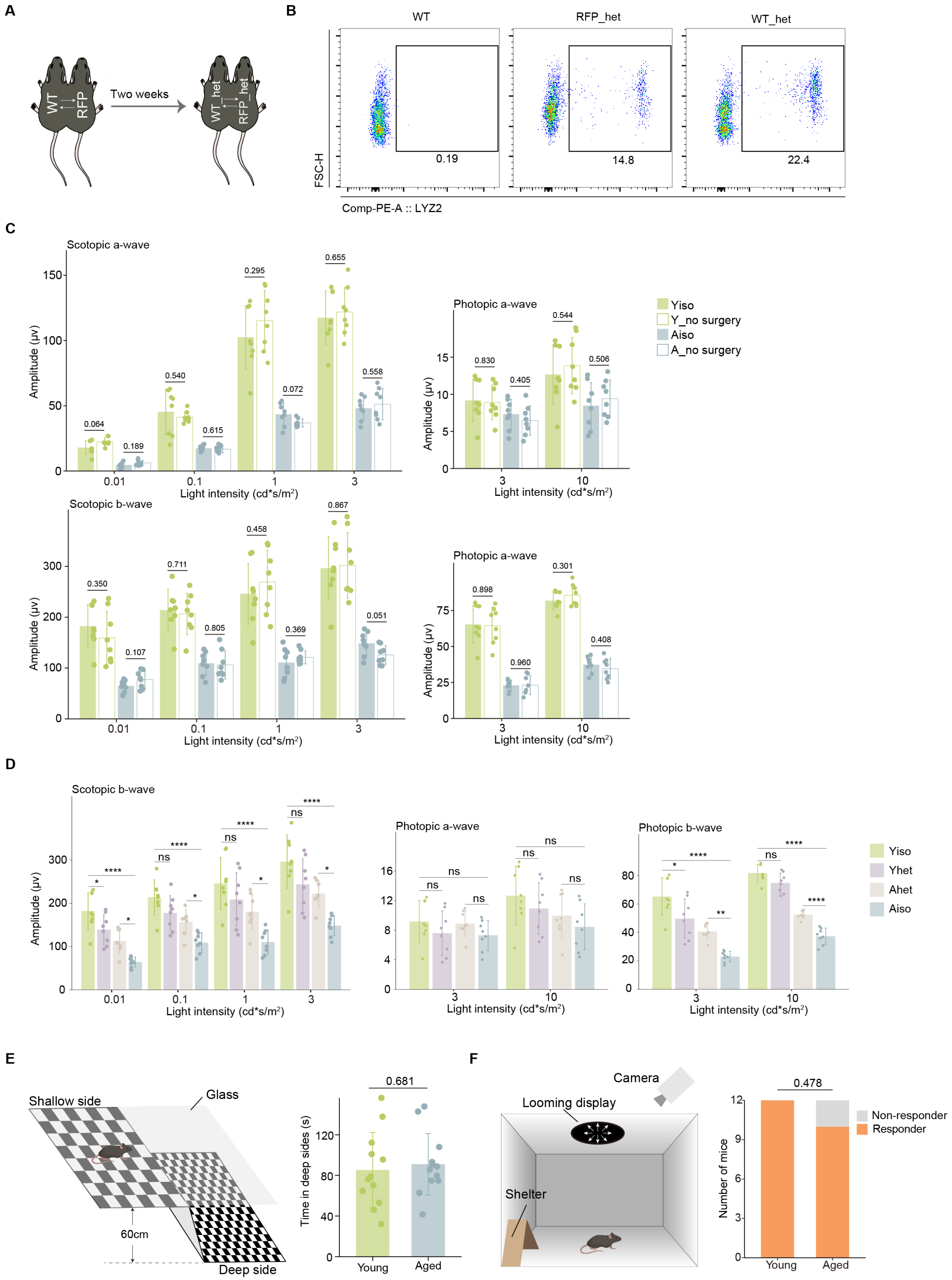
A
WT
RFP
Two weeks
WT_het
RFP_het
B
WT
RFP_het
WT_het
0.19
14.8
22.4
FSC-H
Comp-PE-A :: LYZ2
C
Scotopic a-wave
Amplitude (μv)
0.064
0.189
0.540
0.615
0.295
0.072
0.655
0.558
Light intensity (cd*s/m2)
Photopic a-wave
0.830
0.405
0.544
0.506
Yiso
Y_no surgery
Aiso
A_no surgery
Scotopic b-wave
0.350
0.107
0.711
0.805
0.458
0.369
0.867
0.051
Photopic a-wave
0.898
0.960
0.301
0.408
D
Scotopic b-wave
Photopic a-wave
Photopic b-wave
Yiso
Yhet
Ahet
Aiso
E
Shallow side
Glass
60cm
Deep side
0.681
Time in deep sides (s)
Young
Aged
F
Camera
Looming display
Shelter
0.478
Non-responder
Responder
Number of mice
Young
Aged

Fig. S2.

HP induced changes in retinal function and senescence burden both in the young and aged mice. (A) Schematic illustration of parabiosis of WT and RFP mice. (B) Representative FC histograms showing the frequency of RFP+ cells in the blood of WT, RFP- and WT-parabiotic mice. (C-D) Bar plots showing the quantification of scotopic a-wave, scotopic b-wave, photopic a-wave and photopic b-wave at different light densities across the indicated groups (n=8/group). (E) Schematic diagram of the visual cliff test and quantification of the time mice spent on the deep side (n=12/group). (F) Schematic diagram of the looming visual stimulus test and quantification of the number of mice responding to the looming stimuli (n=12/group). Data are shown as mean ± SD. P values were analyzed using unpaired two-tailed Student's t-test (C, E) or one-way ANOVA with Bonferroni post-hoc test (D) or Fisher's exact test (F); ns, non-significant, *P < 0.05, **P < 0.01, ***P < 0.001, ****P < 0.0001.

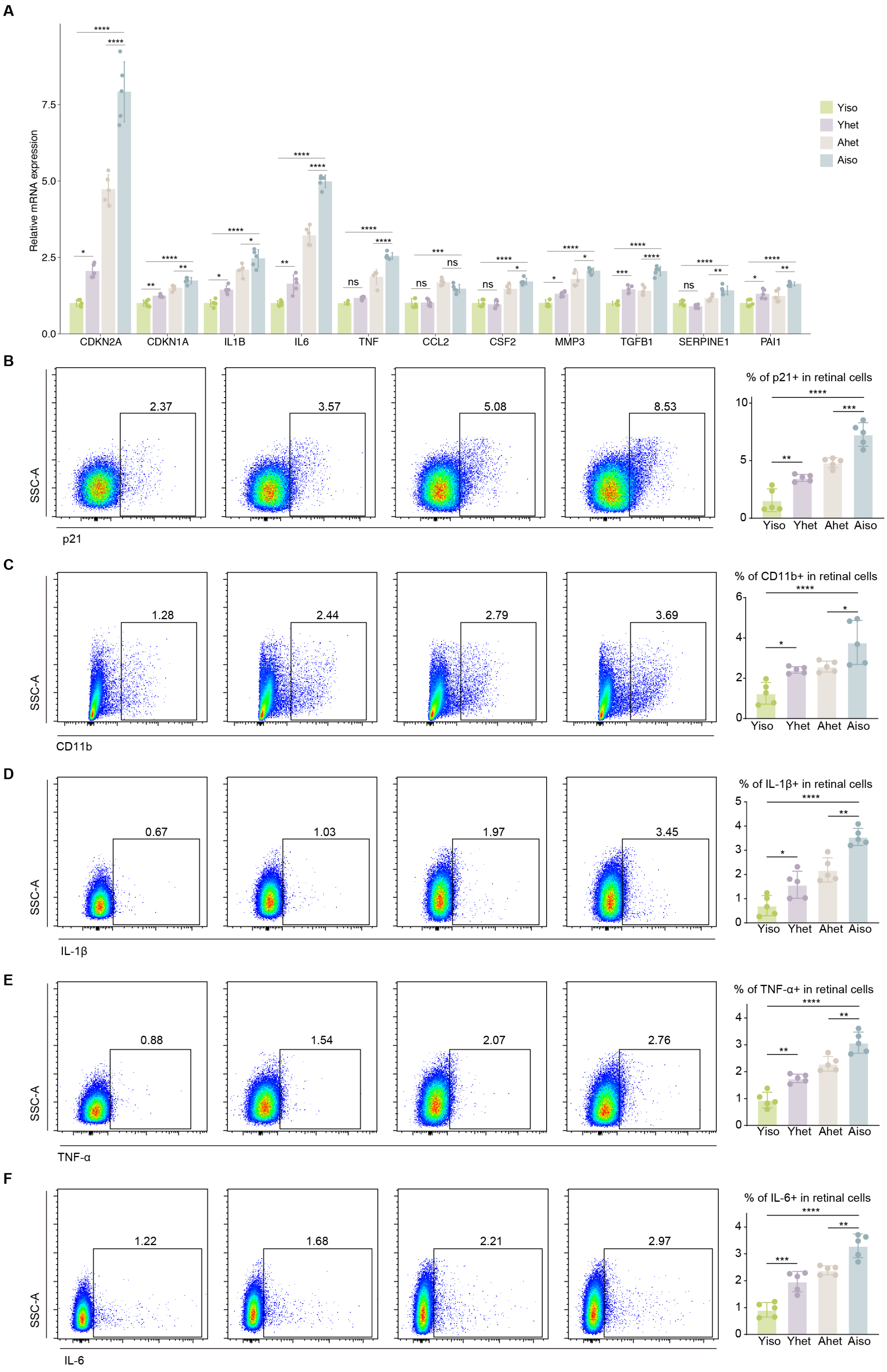
A
B
C
D
E
F
Relative mRNA expression
Yiso
Yhet
Ahet
Aiso
CDKN2A
CDKN1A
IL1B
IL6
TNF
CCL2
CSF2
MMP3
TGFB1
SERPINE1
PAI1
SSC-A
p21
2.37
3.57
5.08
8.53
% of p21+ in retinal cells
CD11b
1.28
2.44
2.79
3.69
% of CD11b+ in retinal cells
IL-1β
0.67
1.03
1.97
3.45
% of IL-1β+ in retinal cells
TNF-α
0.88
1.54
2.07
2.76
% of TNF-α+ in retinal cells
IL-6
1.22
1.68
2.21
2.97
% of IL-6+ in retinal cells

Fig. S3.

HP induced changes in senescence markers and SASP factors both in the young and aged mice. (A) Bar plot showing the relative mRNA expression of senescence markers and SASP factors across the indicated groups (n =5/group). (B-F) FC histograms (left) and column charts (right) showing p21 (B), CD11b (C), IL-1β (D), TNF-α (E), and IL-6 (F) levels in retinal cells across the indicated groups (n = 5/group). Data are shown as mean ± SD. P values were analyzed using one-way ANOVA with Bonferroni post-hoc test (A-F); ns, non-significant, *$P < 0.05$, **$P < 0.01$, ***$P < 0.001$, ****$P < 0.0001$.

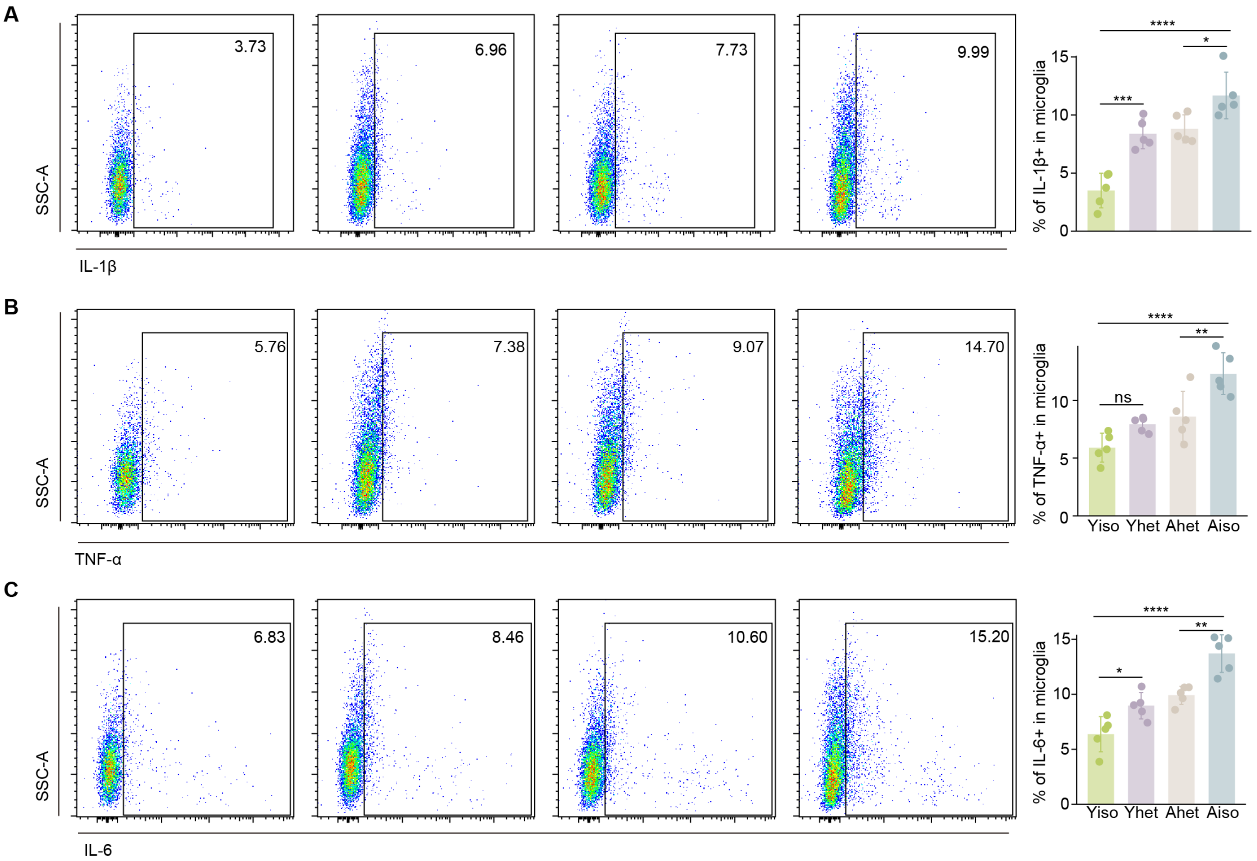

Fig. S4.

HP induced changes in SASP factors in retinal microglia. (A-C) FC histograms (left) and column charts (right) showing IL-1β (A), TNF-α (B), and IL-6 (C) levels in retinal cells across the indicated groups (n = 5/group). Data are shown as mean ± SD. P values were analyzed using one-way ANOVA with Bonferroni post-hoc test (A-F); ns, non-significant, *$P < 0.05$, **$P < 0.01$, ***$P < 0.001$, ****$P < 0.0001$.

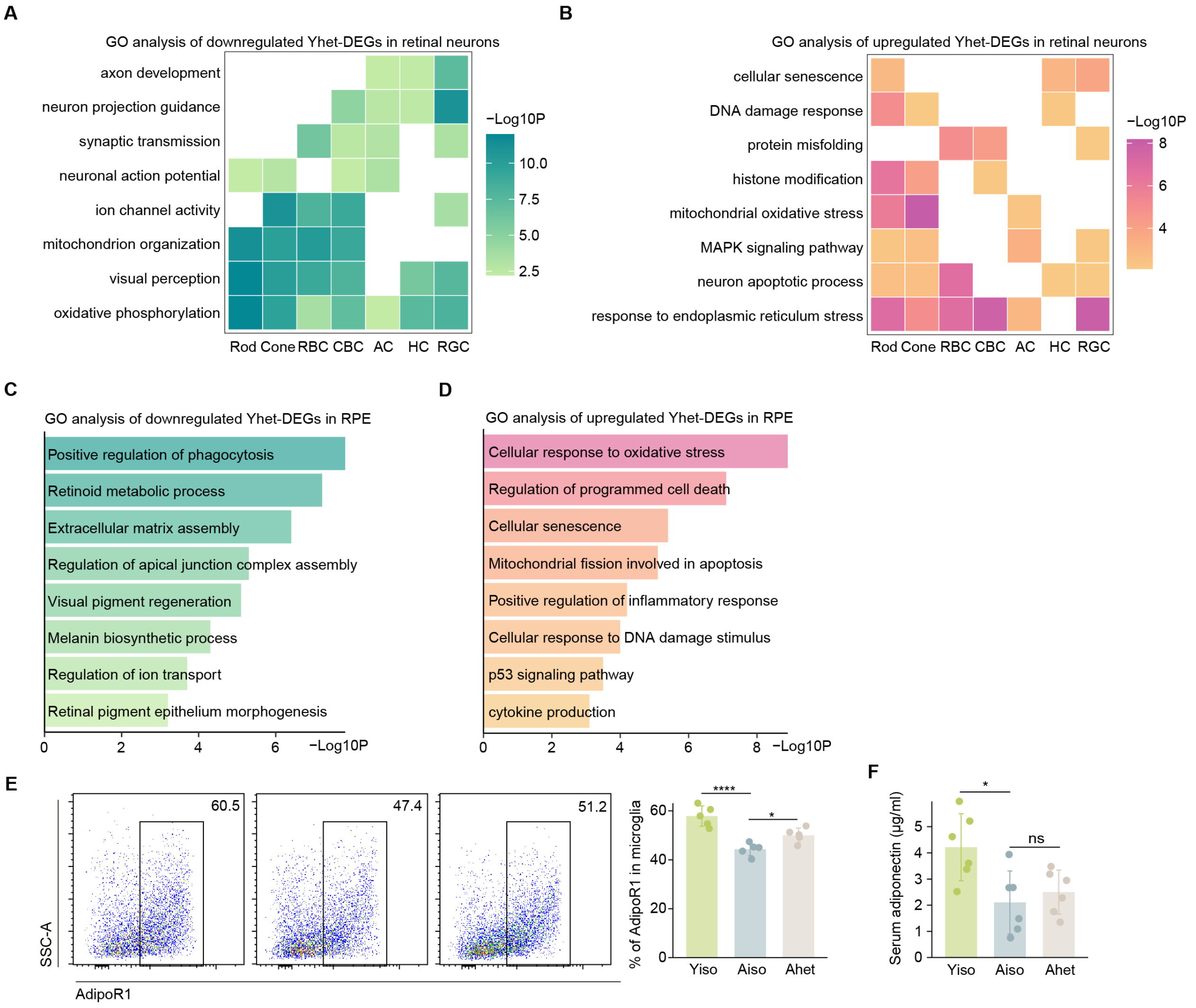

Fig. S5.

Transcriptional alterations across retinal neurons and RPE induced by HP. (A) Heatmap showing GO analysis of downregulated Yhet-DEGs in retinal neurons. (B) Heatmap showing GO analysis of upregulated Yhet-DEGs in retinal neurons. (C) Bar plot showing GO analysis of downregulated Yhet-DEGs in RPE. (D) Bar plot showing GO analysis of upregulated Yhet-DEGs in RPE. (E) FC histograms (left) and column charts (right) showing AdipoR1 levels in microglia across the indicated groups (n = 5/group). (F) Bar plot showing serum adiponectin levels across the indicated groups (n=6/group). Data are shown as mean ± SD. P values were analyzed using one-way ANOVA with Bonferroni post-hoc test; ns, non-significant, *P < 0.05, ****P < 0.0001.

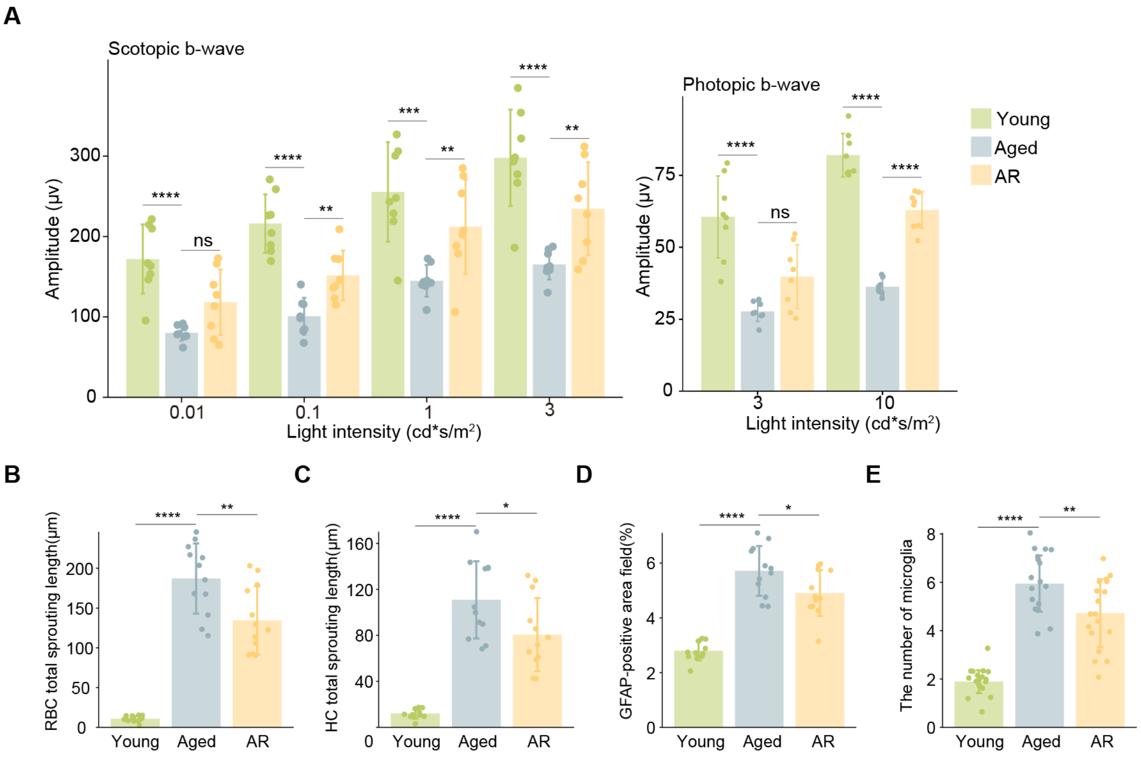

Fig. S6.

AR restored retinal function and structure in the aged mice. (A) Bar plots showing the quantification of scotopic b-wave (left) and photopic b-wave (right) at different light densities across the indicated groups (n = 8/group). (B) Bar plot showing the quantification of RBC total sprouting length across the indicated groups (n = 6/group). (C) Bar plot showing the quantification of HC total sprouting length across the indicated groups (n = 6/group). (D) Bar plot showing the quantification of GFAP-positive area field across the indicated groups (n = 6/group). (E) Bar plot showing the quantification of the number of microglia across the indicated groups (n = 6/group). Data are shown as mean ± SD. P values were analyzed using one-way ANOVA with Bonferroni post-hoc test (A-E); ns, non-significant, *P < 0.05, **P < 0.01, ***P < 0.001, ****P < 0.0001.

A

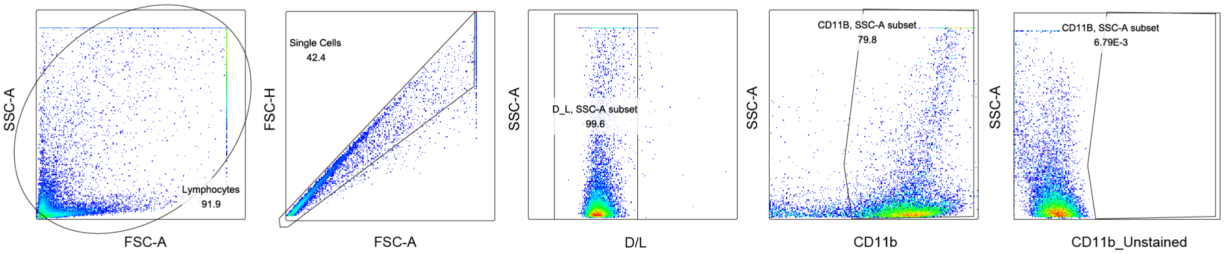

Fig. S7.

AR treatment reduces the senescence burden and alleviates inflammation in primary microglia. (A) Gating strategies for CD11b-positive primary microglia.

	Young	Aged
Rod	6281	6331
Cone	830	732
RBC	408	334
CBC	922	896
AC	578	479
HC	18	17
RGC	28	16
Macroglia	401	435
Microglia	59	107
RPE	604	551
VEC&Pericyte	36	73
Total	10165	9971

Table S1.
Numbers of cells across retinal cell types in each scRNA-seq group in Figure1

	Yiso	Yhet	Ahet	Aiso
Rod	4902	4532	5197	4206
Cone	924	873	789	863
RBC	499	412	426	384
CBC	983	992	737	1031
AC	581	429	506	375
HC	11	5	7	10
RGC	25	22	17	14
Macroglia	435	452	468	506
Microglia	92	121	108	155
RPE	785	836	771	732
VEC&Pericyte	63	32	45	76
Total	9300	8706	9071	8352

Table S2.

Numbers of cells across retinal cell types in each scRNA-seq group in Figure3
